# Cancer-associated fibroblasts actively compress cancer cells and modulate mechanotransduction

**DOI:** 10.1038/s41467-023-42382-4

**Published:** 2023-11-01

**Authors:** Jorge Barbazan, Carlos Pérez-González, Manuel Gómez-González, Mathieu Dedenon, Sophie Richon, Ernest Latorre, Marco Serra, Pascale Mariani, Stéphanie Descroix, Pierre Sens, Xavier Trepat, Danijela Matic Vignjevic

**Affiliations:** 1 Institut Curie, PSL Research University, CNRS UMR 144, F-75005 Paris, France; 2 Translational Medical Oncology Group (ONCOMET), Health Research Institute of Santiago de Compostela (IDIS), University Hospital of Santiago de Compostela (SERGAS), 15706 Santiago de Compostela, Spain; 3 Institute for Bioengineering of Catalonia (IBEC), The Barcelona Institute for Science and Technology (BIST), 08028 Barcelona, Spain; 4 Institut Curie, PSL Research University, CNRS UMR 168, F-75005 Paris, France; 5 Institut Curie, Department of surgical oncology, Curie Institute, F-75005 Paris, France; 6 Facutltat de Medicina, Universitat de Barcelona, 08036 Barcelona, Spain; 7 Institució Catalana de Recerca i Estudis Avançats (ICREA), Barcelona, Spain; 8 Centro de Investigación Biomédica en Red en Bioingeniería, Biomateriales y Nanomedicina (CIBER-BBN), 08028 Barcelona, Spain

**Keywords:** Biophysics, Cell biology, Cancer microenvironment

## Abstract

During tumor progression, cancer-associated fibroblasts (CAFs) accumulate in tumors and produce an excessive extracellular matrix (ECM), forming a capsule that enwraps cancer cells. This capsule acts as a barrier that restricts tumor growth leading to the buildup of intratumoral pressure. Combining genetic and physical manipulations in vivo with microfabrication and force measurements in vitro, we found that the CAFs capsule is not a passive barrier but instead actively compresses cancer cells using actomyosin contractility. Abrogation of CAFs contractility in vivo leads to the dissipation of compressive forces and impairment of capsule formation. By mapping CAF force patterns in 3D, we show that compression is a CAF-intrinsic property independent of cancer cell growth. Supracellular coordination of CAFs is achieved through fibronectin cables that serve as scaffolds allowing force transmission. Cancer cells mechanosense CAF compression, resulting in an altered localization of the transcriptional regulator YAP and a decrease in proliferation. Our study unveils that the contractile capsule actively compresses cancer cells, modulates their mechanical signaling, and reorganizes tumor morphology.

## Abstract (web summary)

Cancer-associated fibroblasts (CAFs) can produce ECM and form a physical barrier around the tumour. Here, the authors show in transgenic mouse models and in vitro systems that CAFs are able to actively compress cancer cells using actomyosin contractility and this leads to a modulation of cancer cell mechanosensing and tumour reorganisation.

## Introduction

Cancer progression is the result of complex interactions between cancer cells and their microenvironment^1^. Cancer cells continuously sense signals from the surroundings that could be either biochemical, such as soluble molecules or membrane receptors on stromal cells, or physical, including stiffness, the microarchitecture of the surrounding extracellular matrix (ECM), fluid pressure and solid stress^2^. Solid stress is a consequence of the proliferation of cancer cells against a viscoelastic boundary, the stroma, which resists tumor growth and prevents its expansion, resulting in the buildup of internal pressure^3,4^.

One of the most abundant cell types in the stroma are cancer-associated fibroblasts (CAFs)^5^. CAFs play multiple roles in cancer progression, promoting cancer cell survival and proliferation and modulating cancer invasion and immune response^5,6^. CAFs are highly contractile cells that serve as the main producers of the ECM^7,8^. Together with the ECM, they form a capsule around the tumor^9,10^. However, whether this capsule is just a barrier that passively opposes tumor growth or has an active role in the generation of tumor stresses remains unknown. In this work we investigated the role of CAFs’ contractility in capsule formation and how CAFs mechanically interact with cancer cells.

## Results and discussion

**Fig. 1:**
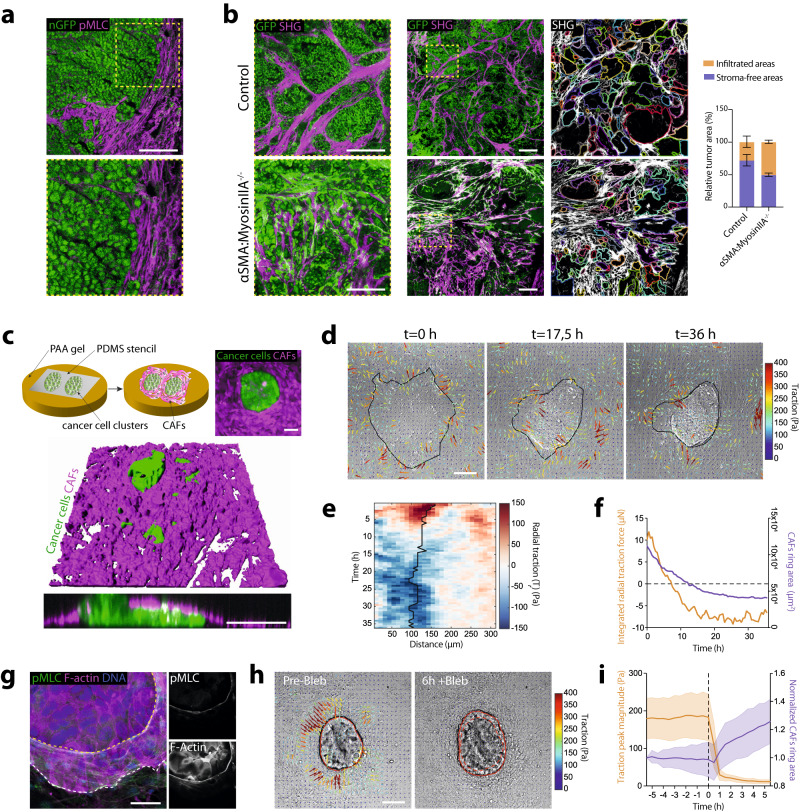
CAFs contractility is required for capsule formation and tumor compartmentalization. **a** Representative (from *n* = 8 positions from *N* = 2 mice) Phospho-Myosin Light chain (pMLC) staining (magenta) in a tumor tissue section from N/p53/mTmG mice. Tumor cells express a nuclear GFP (nGFP). Scale bar 100 µm. **b** Tumors generated after subcutaneous engraftment of N/p53 organoids into control and mice containing myosin IIA-knockout CAFs (quantified in right plot). Middle panel: Cancer cells (nuclear GFP), Myosin IIA KO CAFs (membrane-GFP, green). Collagen I, SHG (magenta). Scale bar: 300 µm. Left panel, magnified boxed regions. Scale bar, 100 µm. Right panel, stroma-free areas based on SHG signal. Right bar chart: quantification of stroma-free (purple) or infiltrated (orange) tumor areas. Data, mean ± SD, *N* = 2 mice/group. **c** Top panel, in vitro co-culture protocol and right panel: representative image (of *N* = 3 independent experiments) of a cluster of primary cancer cells (green) surrounded by CAFs isolated from PDX (magenta). Scale bar, 100 µm. Lower panel, 3D rendering of co-cultures after 48 h. Lower panel, orthogonal Z section. Scale bar: 100 µm. **d** Representative (of *N* = 3 independent experiments) traction maps overlaid on DIC image of cancer cell-CAFs co-culture, evolving over time. Black line-the contour of the cancer cell cluster Scale bar, 100 µm. **e** Representative kymograph of circumferentially averaged radial tractions as a function of the distance to center of the cancer cell cluster (outwards pointing tractions are positive, and inwards pointing tractions negative). Black line-the boundary of the cancer cell cluster. **f** Time evolution of the integrated radial traction force near the boundary of the cancer cell cluster in E (orange) and CAFs ring area (purple). **g** Representative (from *N* = 2 independent experiments) image of CAFs-cancer cells co-culture stained for pMLC (green), F-actin (magenta) and DNA (blue). Insets, magnified boxed region. Scale bar, 100 µm. **h** Traction maps (quantified in panel i) overlaid on a DIC image of cancer cell-CAFs after ≈40 h co-culture (Pre-Bleb) and 6 h after addition of blebbistatin (6 h + Bleb). Solid red line, the contour of CAFs ring; dashed line, CAFs ring contour before blebbistatin. Scale bar, 100 µm. **i** Average radial traction peak magnitude near the cluster cell boundary (orange) and CAFs ring area (purple) normalized to the initial ring size, as a function of time. Dashed black line, blebbistatin addition. Data mean ± SD, *n* = 30 clusters, *N* = 3 independent experiments.

To study the organization of CAFs in tumors, we used a transgenic mouse model that spontaneously develops tumors in the intestinal epithelium due to the expression of an activated Notch1 (NICD) and the deletion of the tumor suppressor p53^10^ (hereafter referred to as N/p53/mTmG) (Supplementary Fig. 1a). Cancer cells were visualized through the expression of a nucleus- and membrane-targeted GFP, while all other cells expressed a membrane-targeted tdTomato. The stroma, composed of CAFs and extracellular matrix (ECM), formed a thick capsule enveloping the tumor. Interestingly, the stroma penetrated the tumor, compartmentalizing it into smaller clusters (Supplementary Fig. 1b; Supplementary Video 1). Each cluster was enwrapped with aligned CAFs forming intratumoral capsules. This was remarkably similar to the typical organization of human colorectal cancers^9^. Given that intratumoral capsules were rich in phosphorylated, thus active, myosin II (Fig. 1a), we wondered whether CAFs contractility could play a role in tumor compartmentalization.

To test this, we transplanted N/p53 tumor organoids into transgenic mice in which we conditionally ablated myosin IIA in αSMA-expressing cells (αSMA-CreER^T2^; R26^mT/mG^; myosin IIA^fl/fl^). In this mouse model, cancer cells expressed nuclear GFP. All stromal cells, including CAFs with wild-type myosin IIA, expressed a membrane-targeted tdTomato. In αSMA-expressing cells, tdTomato switched to GFP concomitantly with the induction of myosin IIA knockout (Supplementary Fig. 2). In control mice, as in spontaneously developed tumors, CAFs generated intratumoral capsules that compartmentalized and confined cancer cells (Fig. 1b). These capsules were largely absent from mice in which CAFs lacked myosin IIA and, instead, cancer cells and CAFs appeared mixed. To quantify this, we segmented large tumor areas using the collagen signal visualized by second harmonic generation (SHG) imaging as a proxy for tumor-stroma boundary. We found that control tumors contained larger stroma-free areas than tumors containing myosin IIA depleted CAFs (Fig. 1b). These data show that CAFs’ contractility is required for the formation of intratumoral capsules, and thus segregation of cancer cells into compartments.

To study the mechanism by which CAFs confine cancer cells, we followed a bottom-up approach and developed an in vitro co-culture system. First, we isolated cancer cells and CAFs from patient-derived xenografts (PDX) of colorectal cancer (Supplementary Fig. 3). As in the tumors, most cultured CAFs expressed αSMA (Supplementary Fig. 3), together with other markers characteristic of myCAFs and iCAFs subpopulations (Supplementary Fig. 4). To mimic tumor organization, we fabricated flat 11 kPa polyacrylamide gels coated with collagen I and we generated circular clusters (150 µm radius) of cancer cells surrounded by CAFs (Fig. 1c). CAFs aligned to each other and parallel to the cancer cell boundary, encapsulating cancer cells and restraining their spreading. Surprisingly, after 8 h, CAFs assembled into a multicellular ring that slid on top of the cancer cells. As the ring advanced, it deformed clusters, ultimately inducing the multilayering of cancer cells and forming a three-dimensional bud (Fig. 1c, Supplementary Video 2). At this point, the ring ceased to advance, and the bud remained stable. CAFs also encapsulated and induced budding of N/53 tumor organoids (Supplementary Fig. 5a–c, Supplementary Video 3). Notably, the same sequence of events was observed ex vivo, where CAFs and cancer cells spontaneously exited from PDX tumor fragments (Supplementary Fig. 5d, Supplementary Video 4). This shows that CAFs can form capsule-like structures to confine cancer cells even in reductionist 2D in vitro environments. The fact that this capsule deforms and reshapes tumor cell clusters suggests that CAFs are not just a passive barrier against cell spreading but rather an active entity that exerts forces on cancer cells.

To understand the mechanics of cancer cell-CAF interaction, we quantified tissue forces using traction force microscopy (TFM) (Fig. 1d, Supplementary Fig. 6, Supplementary Video 5). Forces fluctuated across the tissue but systematically accumulated at the boundary between the CAFs and the cancer cell cluster. Given the radial symmetry of the system, we decomposed tractions into radial (*T*_r_) and tangential (*T*_t_) components, respective to the cluster boundary. We circumferentially averaged these tractions for each timepoint and plotted them as a function of the distance to the cluster center to build kymographs (Fig. 1e, Supplementary Fig. 6b). We found that average tangential forces, which likely correspond to migration of CAFs around cancer cell clusters, remained low and constant over time (Supplementary Fig. 6b, c). In contrast, radial tractions were maximal at the boundary between cancer cells and CAFs (Fig. 1d, e, Supplementary Fig. 6c). Initially, radial tractions were positive (pointing away from cancer cells), probably due to CAFs crawling towards the cluster (Fig. 1d–f). Later, when CAFs aligned parallel to the cluster boundary, radial tractions became negative (pointing towards cancer cells). These negative radial forces progressively increased as CAFs formed the supracellular ring, slid on top of the cancer cells and induced budding, until a point where these tractions stabilize to a plateau value (Fig. 1f). Notably, the same traction pattern was observed on softer substrates (2 kPa) (Supplementary Fig. 7a).

To better understand how CAFs induce the remodeling of cancer cell clusters, we developed a physical model that relates the closure dynamics of the CAF ring and the traction forces to the transmission of stress between CAFs and cancer cells (Supplementary Theoretical Note 1). In the model, CAF closure is assumed to be driven by a purse-string mechanism^11^, which corresponds to a line tension acting at the inner edge of the CAF monolayer. The increase of inward-pointing traction forces as the ring closes reflects the increase of tension at the CAF inner edge, which is, to a first approximation, proportional to the ring curvature for a constant line tension (Supplementary Theoretical Note 1). The observation of inward-pointing traction forces underneath the cancer cells (Fig. 1e) suggests that the CAFs generate shear stress on the cancer cell, modeled as viscous friction between the two layers. We also observed the long-term persistence of traction forces after the ring had stalled (Fig. 1f), which prompted us to adopt an elastic description of the CAFs and cancer cell cluster and an elastic interaction with the substrate. This yields a spatial localization of the traction forces outside the cancer cell cluster, as seen in the experiments (*λ*s~50 μm in Supplementary Theoretical Note 1, Fig. 1b).

The shear stress induced by the CAF motion on top of the cancer cell cluster drives an inward flow of cancer cells and cluster deformation, eventually leading to cancer cell multilayering and bud formation as the CAF ring closes. Bud formation can be seen as a yield-stress problem by considering the cluster of cancer cells as an elastoplastic material where multilayering occurs beyond a critical cluster compression. This model yields a phase diagram for bud formation, which predicts the formation of stable buds - as observed in our experiments - for a limited range of ring line tension (Supplementary Theoretical Note 1, Fig. 1f, g). The model shows that the appearance of the bud can be directly inferred from the integrated traction force, which plateaus prior to ring stabilization (Fig. 1f), indicating that a fraction of the ring tension is resisted by bud compression (Supplementary Theoretical Note 1, Fig. 1d).

Similar to in vivo intratumoral capsules (Fig. 1a), in vitro CAF rings were enriched in phosphorylated myosin II (Fig. 1g). This suggests that ring constriction is driven by actomyosin contractility. Indeed, myosin IIA knockout CAFs exerted significantly lower traction forces than control CAFs (Supplementary Fig. 8a–c) and failed to compress and deform cancer cell clusters even if forced to encircle them due to the experimental setup geometry (Supplementary Fig. 8d, e). Besides a decrease in contractility, myosin IIA knockout CAFs proliferate less than control CAFs (Supplementary Fig. 8f), probably due to the role of Myosin IIA in cytokinesis^12^. Lower CAFs’ proliferation may decrease the compression of cancer cells due to a decreased mitotic pressure independently of contractility. To detangle the contribution of CAFs contractility and mitotic pressure to compression, we inhibited contractility using either blebbistatin to inhibit myosin II (Fig. 1h) or Y27632 to inhibit ROCK (Supplementary Fig. 6d). Both treatments induced a fast relaxation of CAF rings, almost complete disappearance of traction forces, and the flattening of the bud (Fig. 1h, i, Supplementary Fig. 6d, e, Supplementary Video 6). The relaxation of compression was much faster than any expected effect of the drugs on cell proliferation, thus we conclude that CAF contractility is the main driver of compressive forces.

We also tested whether normal fibroblasts could compress cancer cells. Interestingly, while NIH/3T3 were not compressive and exerted tractions pointing away from the cancer cell cluster, intestinal fibroblasts compressed cancer cells even more than CAFs (Supplementary Fig. 7a). Thus, compression is not a CAF-specific ability, suggesting that intestinal fibroblasts (many of those are αSMA^+^ and referred as myofibroblasts^13^) may also compress the surrounding epithelial cells in the healthy gut, as observed in other tissues during development^14^. Indeed, both CAFs and intestinal fibroblasts exerted high tractions at the single cell level, compared to NIH/3T3 (Supplementary Fig. 7b). Overall, our in vitro force measurements and theoretical predictions suggest that CAFs intratumoral capsules not only passively confine cancer cells but also actively compress them.

**Fig. 2:**
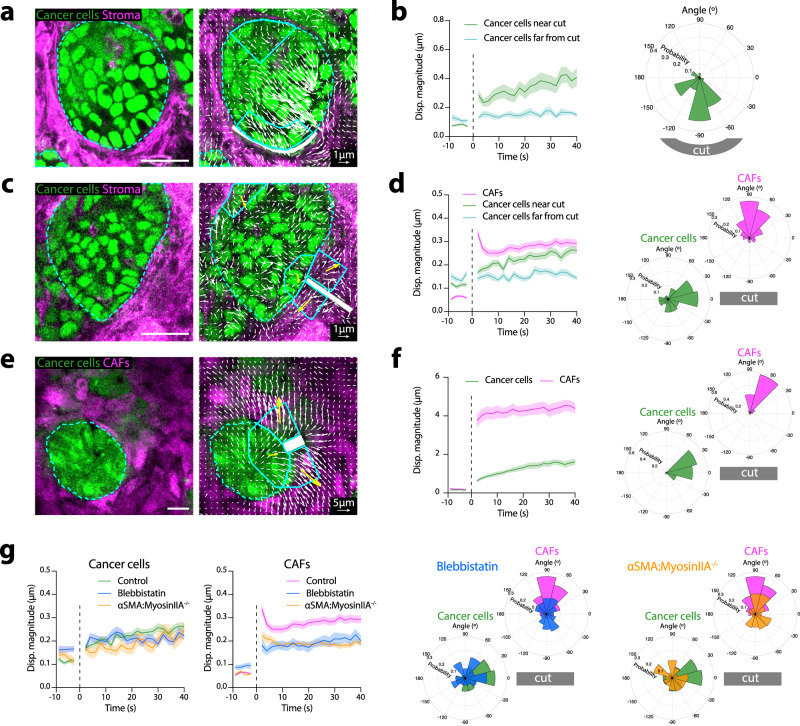
CAFs compress cancer cells using actomyosin contractility. **a** Images before (left) and after laser ablation (right) in fresh tumor slices (quantified in **b**). Cancer cells (green), stroma (CAFs) (magenta). Dashed cyan line - contour of cancer cells. Solid cyan lines delineate ROIs. Laser cut region represented in white. White vectors, tissue displacement 40 s after ablation. Scale bar, 100 µm. Scale vector, 1 µm. **b** Displacement magnitude in ROIs as a function of time after ablation (left). Vertical dashed line (*t* = 0) indicates the ablation time. Polar histogram of displacement angle probability relative to the cut (right) 40 s after ablation. *n* = 31 ablations from *N* = 7 mice. **c**, Montage showing images before (left) and after laser ablation (right) in tumor slices (quantified in panel d). Cancer cells (green), stroma (CAFs) (magenta). Dashed cyan line - contour of cancer cells. Solid cyan lines delineate ROIs. Laser cut region, white. White vectors, tissue displacement maps 40 s after ablation. Yellow vectors, average displacement for each ROI (for visualization purposes, yellow vectors are not scaled). Scale bar, 100 µm. Scale vector, 1 µm. **d** Displacement magnitude of cancer cells and CAFs as a function of time after ablation (left). Vertical dashed line (*t* = 0) indicates the ablation time. Polar histograms of displacement angle probability relative to the cut 40 s after ablation. *n* = 74 ablations from *N* = 7 mice. **e** Montage showing images before (left) and after laser ablation (right) in in vitro co-cultures. Cancer cells (green), CAFs (magenta). Dashed cyan line - contour of cancer cells. Solid cyan lines delineate ROIs. Laser cut region (white box). White vectors, tissue displacement 40 s after ablation. Yellow vectors, average cumulative displacement for each ROI. Scale bar, 100 µm. Scale vector, 5 µm. **f** Displacement magnitude of cancer cells and CAFs as a function of time (left) after ablation. Vertical dashed line (*t* = 0) indicates the ablation time. Polar histograms of displacement angle probability relative to the cut, for CAFs and cancer cells (right) 40 s after ablation. *n* = 13 ablations from *N* = 2 independent experiments **g** Displacement magnitude of cancer cells and CAFs as a function of time after ablation, in control tumor slices, slices treated with blebbistatin or tumor slices contacting Myosin IIA-KO CAFs (left). Polar histograms of displacement angle probability relative to the cut 40 s after ablation. *n* = 74 ablations from *N* = 7 mice (control), *n* = 79 ablations from *N* = 6 mice (Myosin IIA KO), and *n* = 58 ablations from *N* = 5 mice (Blebbistatin). Data represented as mean ± SEM for (**b**, **d**, **f**, **g**).

To analyze the mechanical interactions between CAFs and cancer cells in tumors we performed two-photon laser ablations on ex vivo cultured thick tumor slices. First, we disconnected cancer cells from the surrounding stroma by performing ablations parallel to the edge of cancer cells (Fig. 2a, Supplementary Video 7). Upon ablation, we observed a rapid displacement of cancer cells towards the cut in tumor areas nearby the ablated region, whereas almost no displacement was observed in a distant control region (Fig. 2b). These data show that cancer cells are compressed in tumors. To address the tensional state of the CAFs, we also performed cuts perpendicular to the tumor boundary (Fig. 2c; Supplementary Video 7). Again, cancer cells displaced towards the cut, confirming that they were under compression, whereas CAFs recoiled away from the cut, indicating that they were under tension (Fig. 2d). Similar results were obtained using in vitro co-cultures of cancer cells and CAFs (Fig. 2e, f; Supplementary Video 7). Abrogation of contractility using blebbistatin or knocking out CAFs myosin IIA in tumor tissue slices decreased CAF recoil and directionality upon ablation. The immediate effect of blebbistatin cannot be explained by changes in cell proliferation, ruling out mitotic pressure as the main driver of cancer cell compression in vivo (Fig. 2g; Supplementary Video 8). Furthermore, tumors containing myosin IIA-knockout CAFs exhibited a small displacement of cancer cells that was not directed toward the cut (Fig. 2g, Supplementary Video 8). As in this model cancer cells have WT levels of myosin IIA, this shows that cancer cell compression is a direct consequence of CAFs contractility.

**Fig. 3:**
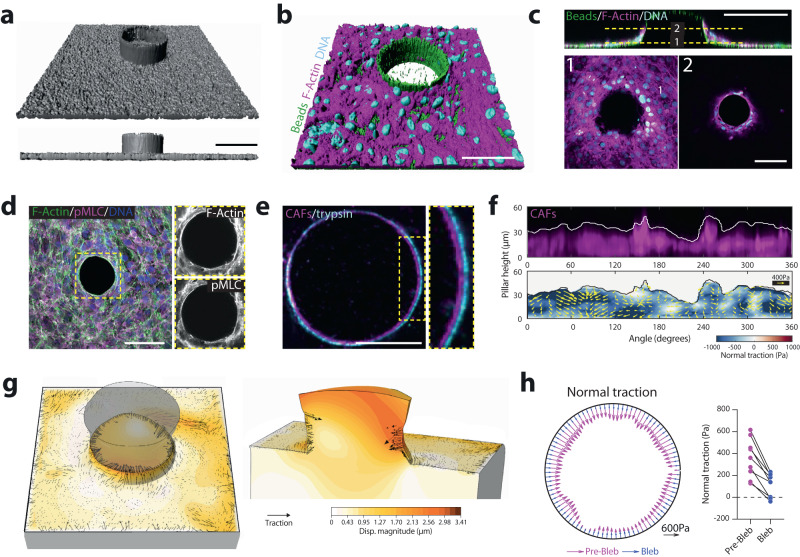
Compression is an intrinsic property of CAFs. **a** PAA pillars containing fluorescent beads (gray). Top, 3D view. Bottom, lateral view. Scale bar, 100 µm. **b** 3D rendering of CAFs stained for F-actin (phalloidin, magenta) and DNA (DAPI, cyan) surrounding a pillar (fluorescent beads, green). Scale bar, 100 µm. *N* = 2 independent experiments. **c** Top, x-z pillar cross-section (fluorescent beads, green) surrounded by CAFs (F-actin, magenta; DNA, cyan). Dashed line represents two selected x-y planes shown at the bottom (1 and 2). Scale bars, 100 µm. *N* = 2 independent experiments. **d** Maximum intensity projection of CAFs surrounding a pillar and stained for F-actin (phalloidin, green), active myosin (pMLC, magenta) and DNA (DAPI, blue). Inset, magnified boxed region showing staining for pMLC and F-actin. Scale bar, 100 µm. **e** Cross-section of a representative (from *n* = 9 pillars, *N* = 2 independent experiments) pillar at its base, in the presence of CAFs (magenta) and after removal of CAFs (trypsin, cyan). Inset, magnified boxed region. Scale bar, 50 µm. **f** Representative unwrapped pillar (from *n* = 9 pillars, *N* = 2 independent experiments) showing CAFs occupancy (top, magenta) and 3D traction force maps (bottom). Color map represents normal tractions (compression is defined as negative, pulling is defined as positive); yellow vectors represent tangential tractions. **g** Representative 3D mapping of deformations (orange) and traction forces (black arrows) exerted by CAFs on a pillar, from *n* = 9 pillars, *N* = 2 independent experiments). Top (left) and side (right) views. Deformations are magnified 5 times for visualization purposes. **h** CAFs normal tractions averaged across pillar height, on a representative pillar before (magenta) and after (blue) blebbistatin treatment. Scale vector, 600 Pa. Right dot plot, quantification of mean normal tractions for each pillar before and after blebbistatin. *n* = 9 pillars, from *N* = 2 independent experiments.

We then asked whether the generation of compressive forces is a CAF intrinsic property or induced by cancer cells. To test this, we microfabricated 50 µm radius soft polyacrylamide pillars (11kPa) to mimic the presence of cancer cells (Fig. 3a, Supplementary Fig. 9a, Supplementary Video 9). CAFs aligned parallel to the pillar border, assembled an actomyosin ring (Fig. 3b–d; Supplementary Video 9) and deformed the pillar (Fig. 3e). We measured pillar deformation using 3D particle image velocimetry and computed CAFs exerted forces using traction force microscopy^15^. This provided 3D traction force fields that we decomposed into a normal component (*T*_n_, perpendicular to the pillar surface) and a tangential component (*T*_*t*_, parallel to the pillar surface). Normal forces exhibited a larger magnitude than tangential forces (~350 Pa vs ~200 Pa on average) and were mostly compressive (negative) (Fig. 3f, g, Supplementary Fig. 9b, Supplementary Video 10). These forces decreased dramatically after inhibition of CAF actomyosin contractility using blebbistatin or Y27632 (Fig. 3h, Supplementary Fig. 9c). Altogether, these data show that CAFs can even compress inert materials and thus, compressive forces emerge from CAFs intrinsic contractility, independently of the presence of cancer cells.

**Fig. 4:**
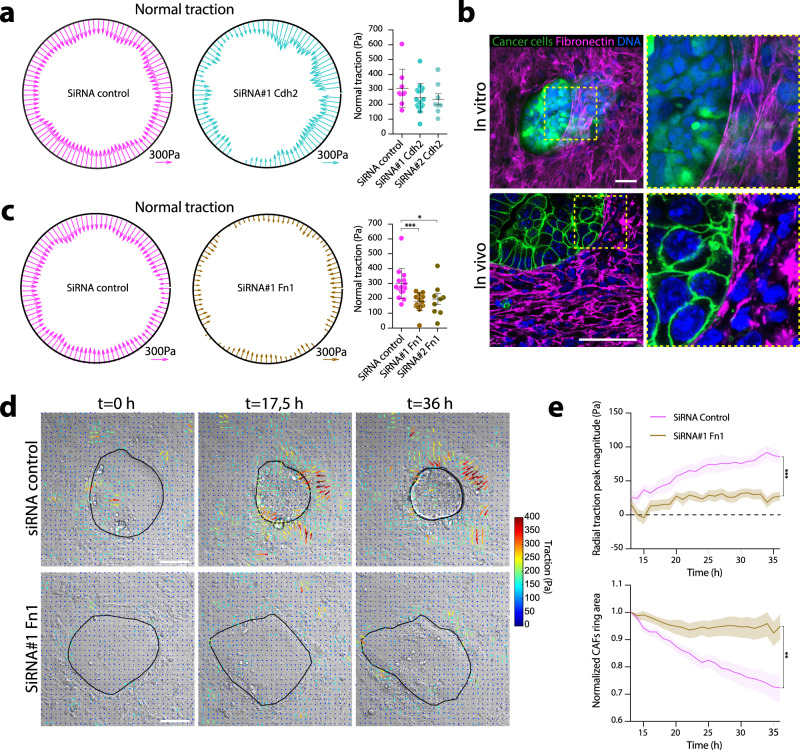
Fibronectin scaffolds allow CAFs supracellular coordination. **a** CAFs normal tractions averaged across pillar height, on a representative pillar for control (magenta) and N-cadherin depleted (cyan) CAFs. Scale vectors, 300 Pa. Right dot plot, quantification of mean normal traction per pillar. Data represented as mean ± SD. *n* = 9 pillars, *N* = 2 (control), *n* = 15 pillars, *N* = 3 (SiRNA#1 Cdh2), and *n* = 8 pillars, *N* = 2 (SiRNA#2 Cdh2). Non-significant, Kruskal-Wallis ANOVA test, Dunns multiple comparisons test. **b** Top left panel, representative image of cancer cells (cell tracker, green) and CAFs in vitro co-cultures (from n = 5 images, *N* = 2 independent experiments), stained for fibronectin (magenta) and DNA (DAPI, blue). Right top panel, inset. Lower left panel, representative image (from *n* = 18 images, *N* = 3 mice) of cancer cells (membrane GFP, green) and CAFs in tumors from N/p53/mTmG mice, stained for fibronectin (magenta) and DNA (DAPI, blue). Scale bars, 50 µm. **c** CAFs normal tractions averaged across pillar height, on a representative pillar for control (magenta) and fibronectin depleted (brown) CAFs. Scale vectors, 300 Pa. Right dot plot, quantification of mean normal tractions per pillar. Data represented as mean ± SD. *n* = 16 pillars, *N* = 4 (control), *n* = 14 pillars, *N* = 4 (SiRNA#1 Fn1), and *n* = 9 pillars, *N* = 2 (SiRNA#2 Fn1). ***p < 0.001, *p < 0.05, Kruskal–Wallis ANOVA test, Dunns multiple comparisons test. **d** Representative (of data plotted in **d**) traction maps overlaid on a DIC image of cancer cell and CAFs control (top) or fibronectin depleted (bottom), evolving over time. Black solid line represents the contour of the CAFs ring. Scale bar, 100 µm. **e** Upper panel, radial traction peak magnitude at the boundary of the cluster for control (magenta) and fibronectin depleted (brown) CAFs, as a function of time. Lower panel, CAFs ring area normalized to the initial cluster size, as a function of time. Data represented as mean ± SEM. *n* = 12 (control) and *n* = 16 (Fibronectin knockdown) clusters, from *N* = 3 independent experiments. ***p < 0.001, **p < 0.01, Mann Whitney two-tailed test for time point *t* = 36.

To effectively compress and deform cancer cells, supracellular CAF rings should be sufficiently stable to maintain integrity over time. What mediates connections between CAFs in the ring? Mesenchymal cells, such as CAFs, generally lack stable cell-cell junctions. Instead, they are interconnected through N-cadherin zipper-like adhesions that allow cell-cell contacts while permitting fast neighbor exchange^16^. To test if N-cadherin mediates connections between CAFs in rings, we depleted N-cadherin using siRNAs (Supplementary Fig. 10a). Surprisingly, we found that N-cadherin-depleted CAFs were still able to compress pillars (Fig. 4a) and exerted the same levels of forces as control cells (Supplementary Fig. 10b).

CAFs produce excessive amounts of ECM proteins, especially fibronectin^9,17–19^. Fibroblasts can use fibronectin to connect to each other using specialized cell-cell contacts, named stitch adhesions^20^. This led us to hypothesize an alternative mechanism for CAF supracellular coordination indirectly via ECM. We observed that CAFs deposited abundant amounts of fibronectin both in vitro and in vivo (Fig. 4b). Consistent with the fact that CAFs contractility is required for fibronectin fibrillogenesis^17^, myosin IIA-knockout CAFs produced lower amounts of fibronectin (Supplementary Fig. 10c, d). The fibronectin network was isotropic and disorganized in the bulk of CAFs but became aligned at the boundary with cancer cells forming supracellular cables that spanned several CAFs (Fig. 4b). Similar reorganization of the fibronectin network was previously seen in fibroblasts growing in macroscopically engineered clefts^21^. This led us to hypothesize that fibronectin could act as a mechanical scaffold for CAF supracellular coordination and force generation during compression. Indeed, the depletion of fibronectin in CAFs significantly reduced their ability to compress pillars (Fig. 4c). Similarly, in the absence of fibronectin, CAFs could not stabilize multicellular rings, which led to a reduction in the radial traction peak when co-cultured with cancer cells (Fig. 4d, e, Supplementary Fig. 10f, Supplementary Video 11). Importantly, this loss of tractions is not due to impaired force generation because control and fibronectin-depleted CAFs exerted similar tractions when cultured as single cells (Supplementary Fig. 10e). Furthermore, the proliferation of fibronectin-depleted CAFs was unaffected (Supplementary Fig. 10g). This shows that fibronectin is dispensable for force generation but required for intercellular force transmission. A direct consequence of this mechanical hindrance was the inability of CAFs to compress cancer cells (Fig. 4d, e). Altogether, these data show that fibronectin cables support force transmission between CAFs, allowing the mechanical coordination required to compress cancer cells.

**Fig. 5:**
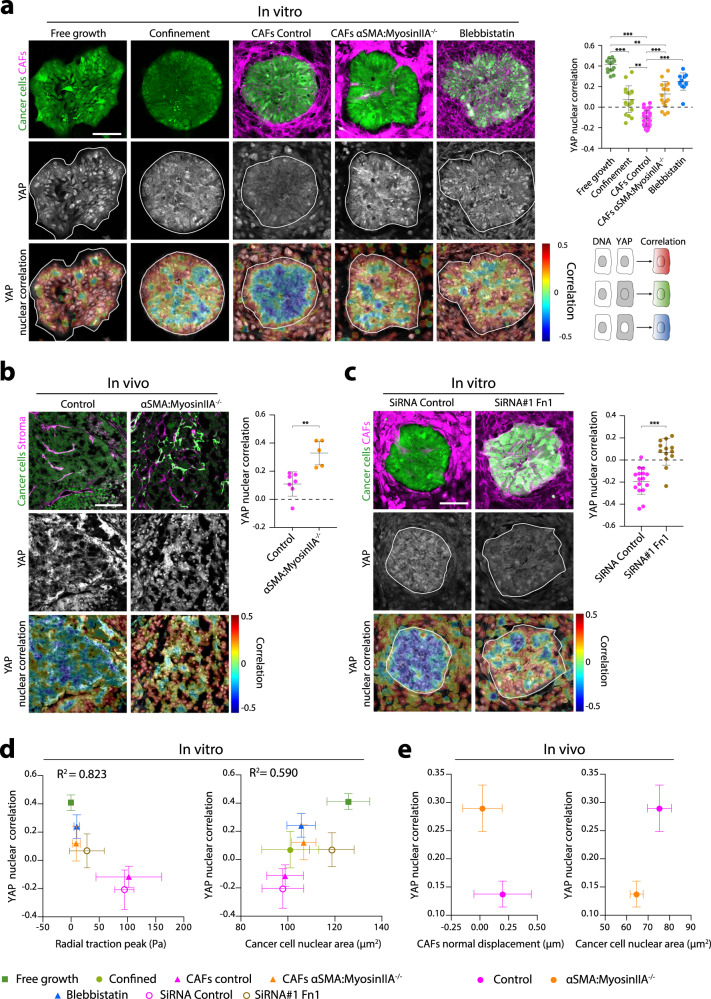
CAFs compression of cancer cells triggers YAP nuclear exit. **a** Patterned cancer cells (Cell tracker, Green) cultured on a collagen-coated polyacrylamide gel growing freely, under confinement (by a PDMS stencil), surrounded by control or Myosin IIA KO CAFs (F-Actin, phalloidin, magenta), or treated with blebbistatin (quantified in right dot plot). Cells are stained for YAP. Right upper panel: YAP nuclear correlation ranging from 0.5 (red, high correlation) to −0.5 (blue, anti-correlation). Lower panel: schematic representation of how correlations are performed. White line outlines the boundary of the cancer cell cluster. Scale bar, 100 µm. Right dot plot: mean YAP nuclear correlation of cancer cells in vitro. Data represented as mean ± SD. Free growth (*n* = 20 clusters, *N* = 5), confinement (*n* = 19 clusters, *N* = 5), CAFs control (*n* = 42 clusters, *N* = 10), CAFs αSMA:Myosin IIA^−/−^ (*n* = 17 clusters, *N* = 4), Blebbistatin (*n* = 12 clusters, *N* = 3). **p < 0.01, ***p < 0.001, Kruskal–Wallis ANOVA test, Dunns multiple comparisons test. **b** Tumors from control or αSMA:Myosin IIA KO mice showing cancer cells (nuclear-GFP, green), CAFs expressing myosin IIA (membrane-tdTomato, magenta) and myosin IIA KO CAFs (membrane-GFP, green), stained for YAP (yellow) and DNA (DAPI, blue). Bottom panel, YAP nuclear correlation. Scale bar, 100 µm. Right dot plot: mean YAP nuclear correlation in cancer cells in vivo. Data represented as mean ± SD, each dot is the average of 3–6 image quantifications per tumor of *n* = 7 control and *n* = 6 myosin IIA KO mice, from *N* = 2 independent experiments. **p < 0.01, Mann Whitney two-tailed test. **c** Cancer cells (Cell tracker, Green) surrounded by CAFs treated with SiRNA Control and SiRNA fibronectin. Scale bar, 100 µm. Right dot plot: mean YAP nuclear correlation in cancer cells. Data represented as mean ± SD, *n* = 12 (SiRNA Control), *n* = 13 (SiRNA Fibronectin) clusters per condition, from N = 3 independent experiments. ***p < 0.001, Mann Whitney two-tailed test. **d** Left graph: Average radial traction peaks plotted against YAP nuclear correlation for all conditions. Right graph: Cancer cell nuclear area plotted against YAP nuclear correlation for all conditions. Correlations were tested by linear regression *(p* < 0.05). Data represented as mean ± SD. *n* and *N* values correspond to the ones in (**a**, **c**). **e** Left graph: CAF normal displacement upon ablation (respect to the ablation) plotted against YAP nuclear correlation in control and αSMA:MyosinIIA^−/−^ tumors. Right graph: Cancer cell nuclear area plotted against YAP nuclear correlation in control and αSMA:MyosinIIA^−/−^ tumors. Data represented as mean ± SD. *n* and *N* values correspond to the ones in (**b**).

To address if cancer cells sense and functionally respond to CAF compression, we analyzed the cellular localization of a well-established mechanosensor, the transcriptional co-activator YAP, which shuttles in and out of the nucleus depending on the mechanical stress to which cells are subjected^22,23^. First, we characterized the mechanosensing ability of the cancer cells by seeding them on soft (0.2 kPa) and stiff (11 kPa) substrates. As observed in other cell types^24^, YAP nuclear to cytoplasmic ratio was increased on stiff substrates, and this effect was abolished upon inhibition of contractility using blebbistatin (Supplementary Fig. 11). To measure YAP localization at the collective level in cancer cell clusters, we quantified the 3D correlation between DAPI and YAP signals (Fig. 5a). A positive correlation indicates that YAP is preferentially in the nucleus, whereas a negative correlation reflects YAP cytoplasmic localization. We found that, in the absence of CAFs, YAP was mostly nuclear in cancer cells growing without any constraint (Fig. 5a). Spatial confinement of cancer cells on micropatterns lead to an increase in cytoplasmic YAP. Upon compression by CAFs, YAP localization in cancer cells was even more cytoplasmic. Lack of CAFs compression, either by inhibition of contractility (blebbistatin and myosin IIA KO) (Fig. 5a, b) or supracellular coordination (Fibronectin KD) (Fig. 5c), triggered the nuclear translocation of YAP to levels comparable to confinement. This shows that contractility-driven compression promotes YAP nuclear exit to levels that cannot be achieved by passive confinement or low substrate stiffness.

YAP nuclear exclusion may be driven by a specific subpopulation of CAFs that is lost upon perturbations in contractility or fibronectin expression. To exclude this possibility, we performed RNA sequencing of Myosin IIA KO and Fibronectin KD CAFs and found that the expression of CAF subtype markers was largely unaffected (Supplementary Fig. 12).

To further understand the mechanism triggering YAP localization, we quantified cell density and nuclear area across all conditions. We used the CAF radial traction peak (in vitro) and the CAF displacement upon ablation (in vivo) as readouts of compression. Both in vitro and in vivo, compression correlated with an increase in cancer cell density (Fig. 5d, e, Supplementary Fig. 13b), a decrease in cancer cell nuclear area (Fig. 5d, e) and the nuclear exclusion of YAP (Fig. 5d, e). Importantly, CAF density did not correlate with YAP localization, again ruling out CAFs mitotic pressure as the main source of compression (Supplementary Fig. 13a). Overall, these data suggest that CAF compression triggers cancer cell packing, decreasing nuclear tension and inducing YAP nuclear export. This hypothesis aligns with previous findings indicating that pulling forces exerted on the nucleus can open nuclear pores and allow YAP to enter the nucleus^22,25,26^.

**Fig. 6:**
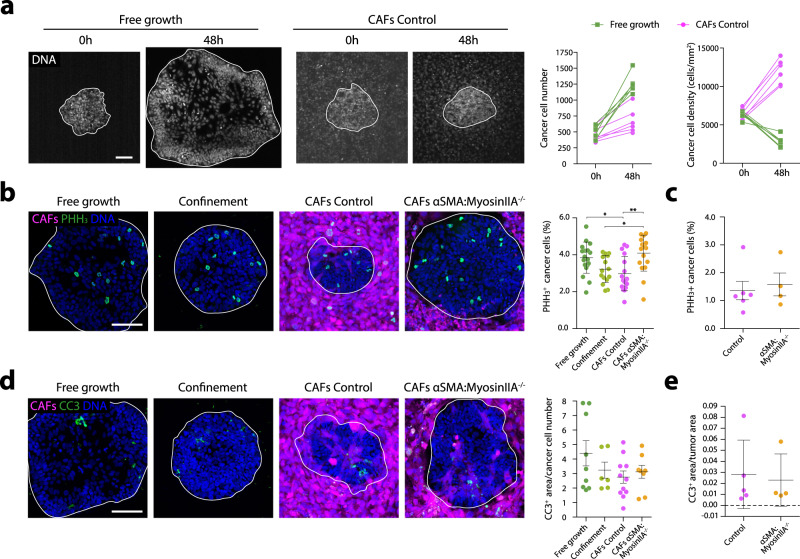
CAFs compression restrains cancer cell growth. **a** Evolution of cancer cell clusters that freely grow (left) or grow compressed by CAFs (right). Cell nuclei are labeled with Hoestch to allow live imaging. White line outlines the boundary of the cancer cell cluster. Graphs: Quantification of the evolution of cancer cell number (left) and density (right). *n* = 6 clusters per condition from *N* = 2 independent experiments. **b** Immunostaining of cells in M-phase (labeled by Phospho-histone H3) in cancer cell clusters that are freely growing, confined or surrounded by control or MyosinIIA KO CAFs. Graph: quantification of the percentage of PHH3^+^ cells in each condition. Data are presented as mean ± SD, *n* = 18 (free growth), 15 (confined), 17 (CAFs control), and 17 (CAFs Myosin IIA KO), from N = 3 independent experiments. *p < 0.05, **p < 0.01, Kruskal–Wallis ANOVA test, Dunns multiple comparisons test. **c** Quantification of percentage of PHH3^+^ cells in control and αSMA:MyosinIIA^-/-^ tumors. Data are presented as mean ± SD, n = 6 (Control), 4 (αSMA:MyosinIIA^−/−^), from N = 1 experiment. Not significative, Mann Whitney two-tailed test. **d** Immunostaining of apoptotic cells (labeled by cleaved-caspase 3) in cancer cell clusters that are freely growing, confined or surrounded by control or MyosinIIA KO CAFs. Graph: quantification of CC3+ area normalized to the total number of cancer cells per cluster. Data are presented as mean ± SD, *n* = 9 (free growth), 6 (confined), 11 (CAFs control), and 8 (CAFs Myosin IIA KO), from *N* = 2 independent experiments. **e** Quantification CC3+ area in control and αSMA:MyosinIIA^−/−^ tumors. Data are presented as mean ± SD, *n* = 5 (Control), 4 (αSMA:MyosinIIA^−/−^), from *N* = 1 experiment. Representative examples of immunostainings quantified in **c** and **e** can be found in Supplementary Data Fig. 15. Scale bars = 100 µm.

We next investigated the functional implications of CAFs compressing cancer cells. We observed that the net number of cancer cells increased at a much slower rate in compressed cancer cell clusters compared to freely growing ones (Fig. 6a). In turn, cancer cell density increased upon compression, while it decreased during cancer cell-free growth (Fig. 6a). These findings suggest CAF compression affects cancer cell growth, either by reducing their proliferation or by increasing apoptosis. In vitro, cancer cell proliferation decreased upon CAFs compression and recovered in the presence of non-contractile myosinIIA-knockout CAFs based on Edu pulses and Ki67 and Phospho-histone H3 (PHH3) staining. In contrast, confinement only decreased the number of mitotic cells (PHH3) (Fig. 6b, Supplementary Data Figs. 14a, 15a). Importantly, conditioned media either from WT or myosin IIA-knockout CAFs did not affect cancer cell growth (Supplementary Fig. 15b), excluding indirect effects on cancer cell proliferation through CAFs nutrient consumption^27^. In contrast, cancer cell apoptosis was not affected by compression (Fig. 6d). We thus conclude that CAFs compression restricts cancer cell proliferation, probably through YAP mechanosensing^28^, although other mechanisms may also be involved. In fact, cancer cell proliferation and apoptosis were not affected in tumors containing myosin IIA-knockout CAFs, despite changes in YAP localization (Fig. 6c, e, Supplementary Figs. 14b, 16). The discrepancy between in vitro and in vivo results could be due to additional factors regulating cancer cell proliferation in tumors that are absent in our simplified in vitro model, such as cancer cell access to nutrients or stroma-secreted factors.

Overall, we found that CAFs assemble intratumoral capsules stabilized by fibronectin scaffolds, allowing coordinated supracellular contraction. These contractile capsules actively compress cancer cells, triggering mechanical signaling and inducing reorganization of the tumor architecture. Thus, in contrast to the generally accepted concept, the tumor capsule is not just a passive barrier that prevents tumor expansion, and our data rather points towards capsules as active determinants of tumor solid stress. Although these results need to be validated on more complex in vitro 3D environments, our findings could have diverse effects on tumor progression^29^. It could slow down tumor growth but, at the same time, promote cancer cell stemness^30^, drug-resistance^31^, collapse of blood vessels^4^ and metastatic traits by increasing DNA damage^32^, as well as tumor budding, a poor prognosis factor for colorectal cancer patients^33–36^. If those processes occur simultaneously or at different stages of tumor progression remains to be understood, and new mouse models coupled with advanced real-time in vivo imaging techniques would help to unambiguously ascertain the effects of CAF compression on tumor progression.

## Methods

### Ethical statement

Animal care and use for this study were performed in accordance with the recommendations of the European Community (2010/63/UE) for the care and use of laboratory animals. Experimental procedures were specifically approved by the ethics committee of the Institut Curie CEEA-IC #118 (Authorization NUMBER −25603-2020053122444776- given by National Authority) in compliance with the international guidelines.

### Mouse models

All mice were kept under Specific Pathogen Free (SPF) conditions for breeding. Double fluorescent pVillin-CreER^T2^, LSL-NICD-nGFP; mT/mG; p53^fl/fl^ mice were generated as previously described^10^. Four-weeks old male and female mice were injected intraperitoneally with tamoxifen (50 mg/kg) for 5 consecutive days for induction of Cre recombinase activity. Approximately 8 months after tamoxifen injection, mice spontaneously develop invasive intestinal carcinomas^37^. All cells from these mice express a membrane-targeted tdTomato, while tumor cells express a nuclear-targeted GFP.

Double fluorescent SMA-CreER^T2^; R26^mT/mG^; myosin IIA^fl/fl^ were generated by crossing mice containing the MyoIIA heavy-chain (*Myh9*) floxed^38^ with mice expressing Cre recombinase under the control of the SMA promoter^39^, and with Rosa26-mTmG^40^ mice. Rosa26-mTmG mice express a membrane-targeted tandem dimer Tomato (mT) prior to Cre-mediated excision and a membrane-targeted GFP (mG) following excision. Membrane targeting was achieved using the MARCKS membrane tag.

### Generation and culture of tumor organoids

Tumors from pVillin-CreER^T2^, LSL-NICD-nGFP; mT/mG; p53^fl/fl^ male and female mice were excised and dissociated using a scalpel in medium containing DMEM-F12 (ThermoFisher Scientific) supplemented with 2,5% (v/v) GlutaMAX (Gibco), 2% (v/v) Antibiotic-Antimycotic (Gibco) and 300 units/ml of Collagenase III (StemCell). Tissue pieces were incubated for 2 h at 37 °C under agitation (180 rpm), and then filtered first through 100 µm and 40 µm filters. Dissociated cells were centrifuged and resuspended in a 100 µl drop containing a mix of 50% Matrigel (Corning)− 50% (v/v) tumoroid medium (DMEM-F12, supplemented with 2,5% (v/v) GlutaMAX, 2% (v/v) antibiotic-antimycotic, 100 ng/mL Noggin (Peprotech), 50 ng/mL EGF (Peprotech), 10 ng/mL (Peprotech), 1% (v/v) B27 supplement (ThermoFisher Scientific) and 1% (v/v) N-2 supplement (ThermoFisher Scientific)). Matrigel drops were allowed to polymerize for 40 min at 37 °C and 5% CO_2_ and were then covered with 2 mL of tumoroid medium. Once formed, tumoroids were split once a week.

### Tumor establishment in αSMA MyoIIA KO mice

Bone marrow transplantation experiments were performed in order to render the immune system of αSMA MyoIIA KO mice compatible with tumor growth.

#### Irradiation

6–8 weeks old recipient male and female mice (SMA-CreER^T2^; R26^mT/mG^; myosin IIA^fl/fl^) were placed in an acrylic container in continuous airflow between two opposite X-ray sources (CIXD Dual Irradiator, Xstrahl). Cre^−/−^ mice were used as controls. Mice were exposed to a Fractioned Total Body Irradiation (FTBI), at a rate of 1,18 Gy/min for a total dose of 10 Gy, fractionated in two 5 Gy doses with a 4 h interval.

#### Bone marrow transplantation

Donor mice (non-induced pVillin-CreER^T2^, LSL-NICD-nGFP; mT/mG; p53^fl/fl^, 4-10 months old, male and female) were sacrificed and bone marrow cells (BMCs) were collected from the tibias, femurs and humerus, and resuspended in 300 µl of PBS containing 2% (v/v) FBS. Immediately after irradiation, sex-matched recipient mice were injected retro-orbitally with 100 µl of the BMCs suspension, containing approximately 2 × 10^6^ BMCs, using a 27-gauge needle. Mice were left for 8 weeks to allow efficient grafting of BMCs and reconstitution of the immune system.

#### Tumor establishment and MyosinIIA KO induction

Tumoroids from pVillin-CreER^T2^, LSL-NICD-nGFP; mT/mG; p53^fl/fl^ mice were cultured as described above. Prior to injection in mice, tumoroids from 12 24-well Matrigel drops were harvested and mechanically disaggregated using a pipette tip, centrifuged and resuspended in 100 µl of 1:1 Matrigel/culture medium. This solution was injected into the interscapular fat pad of bone marrow transplanted mice. For this, under Isoflurane gas anesthesia, a small skin incision was performed at the level of the interscapular region, the fat pad was exteriorized and tumoroids were injected directly into it using a 25 G needle. Mice were then sutured using wound clips (7.5*1.5 mm, Phymep), and placed into standard housing conditions during the time of tumor development. 3 weeks post engraftment mice were injected daily with tamoxifen (50 mg/kg) for two consecutive days, to induce Cre-mediated gene recombination. Tamoxifen was re-administered either every week or every other week throughout the entire duration of the experiment to ensure efficient knockout of possibly newly generated CAFs. For tumor compartmentalization analyses we used mice injected every week with tamoxifen. For laser ablation experiments, all mice were analyzed, as ablations were performed specifically in areas with high content of knockout CAFs (mGFP^+^) To account for possible secondary effect of tamoxifen in tumor development, all mice (Cre^+/−^ and Cre^−/−^ controls) were injected with the same doses of tamoxifen. 9 weeks after tumoroid injection mice were sacrificed, and tumors were excised and prepared for downstream analysis.

### Immunostaining of tumor tissue sections

Tissue was fixed in 4% paraformaldehyde (Electron Microscopy Sciences)/PBS (v/v) for 20 min at RT, and then dehydrated first in 15% sucrose (Sigma-Aldrich)/PBS (w/v) solution for 1 h and then in 30% sucrose/PBS (w/v) solution for 2 h at RT. After, tissue was embedded with OCT compound (Sakura) in plastic gaskets (Euromedex), frozen at −20 °C and cut on the cryostat using SuperFrost Plus™ Adhesion slides (VWR, Menzel Gläser). Tissue sections were then permeabilized with 0.2% Triton x100 (Sigma-Aldrich)/PBS (v/v) for 1 h at RT, blocked with 3% BSA (w/v) (IgG-Free, Protease-Free, Jackson Immuno Research) in 0.05% Triton x100/PBS (v/v) solution for 1 h at RT and stained with primary antibodies overnight in humidified chambers at RT. Sections were then washed 3 times with 0.05% Tx100/PBS (v/v) solution for 1 h, incubated with secondary antibodies, DAPI and phalloidin, depending on the staining, for 4 h at RT, washed 3 times in 0.05% Tx100/PBS (v/v) solution for 1 h and mounted using AquaPolyMount (Polysciences). Antibodies references and dilutions are listed in Table 1.

**Table 1:** Antibodies used in the study

Antigen	Antibody/Chemical	Dilution					Reference	Vendor
		FACS	Cryosections	Whole-mount	Cultured cells	Western blot		
Fibronectin	Anti-Fibronectin rabbit polyclonal antibody	–	1–100	1–100	1–200	1–10,000	F3648	Sigma Aldrich
N-Cadherin	Anti-N-Cadherin mouse monoclonal antibody	–	–	–	–	1–500	33-3900	Thermo Fisher Scientific
MyosinIIA	Anti-MyosinIIA rabbit polyclonal antibody	–	–	1–100	1–100	1–100	909801	Biolegend
GAPDH	Anti-GAPDH rabbit polyclonal antibody	–	–	–	–	1–5000	G9545	Sigma Aldrich
αSMA	Anti-αSMA mouse monoclonal antibody	1–200	–	–	1–100	–	A2547	Sigma Aldrich
pMLC	Anti-pMLC rabbit polyclonal antibody	–	–	1–100	1–200	–	3674	Cell Signaling
YAP	Anti-YAP rabbit monoclonal antibody	–	1–200	-	1–200	–	14074	Cell Signaling
CC3	Anti- Cleaved Caspase 3 rabbit monoclonal antibody	–	-	1–100	1–200	–	9661	Cell Signaling
PHH3	Anti-Phospho Histone H3 rabbit monoclonal antibody	–	1– 200	–	1–200	–	H0412	Sigma Aldrich
Ki67	Anti-Ki67 rabbit monoclonal antibody	–	1–200	–	1–400	–	9129S	Cell Signaling
EpCAM	PE/Cy7 anti-human CD326, mouse IgG2b, κ	1–10	–	–	–	–	324221	Biolegend
EpCAM	APC anti-mouse CD326, mouse, rat IgG1	1–10	–	–	–	–	130-102-969	Miltenyi Biotec
CD45	Brilliant-Violet 421 anti-human CD45, mouse IgG1, κ	1–20	–	–	–	–	304032	Biolegend
CD45	Brilliant-Violet 421 anti-mouse CD45, rat IgG2b, κ	1–20	–	–	–	–	103133	Biolegend
CD31	FITC anti-human CD31, mouse IgG1, κ	1–20	–	–	–	–	303104	Biolegend
CD31	FITC anti-human CD31, rat IgG2a, κ	1–20	–	–	–	–	102405	Biolegend
Vimentin	APC anti-human Vimentin, recombinant IgG1	1–250	–	–	–	–	130-106-370	Miltenyi Biotec
Isotype controls	FITC mouse IgG1	1–10	–	–	–	–	130-092-213	Miltenyi Biotec
	Brilliant-Violet 421 rat IgG2b, κ	1–20	–	–	–	–	400639	Biolegend
	FITC rat IgG2a, κ	1–20	–	–	–	–	400505	Biolegend
Anti-mouse Alexa Fluor 488	Goat anti-mouse IgG Alexa Fluor 488 polyclonal antibody	–	1–200	1–200	1–400	–	A11029	Thermo Fisher Scientific
Anti-mouse Alexa Fluor 546	Goat anti-mouse IgG Alexa Fluor 546 polyclonal antibody	–	1–200	1–200	1–400	–	A11030	Thermo Fisher Scientific
Anti-Rabbit Alexa Fluor 488	Goat anti-Rabbit IgG Alexa Fluor 488 polyclonal antibody	–	1–200	1–200	1–400	–	A32731	Thermo Fisher Scientific
Anti-Rabbit Alexa Fluor 568	Goat anti-Rabbit IgG Alexa Fluor 568 polyclonal antibody	–	1–200	1–200	1–400	–	A11011	Thermo Fisher Scientific
Anti-rabbit HRP	Goat anti-Rabbit polyclonal antibody						32260	Thermo Fisher Scientific
	Against Fibronectin	–	–	–	–	1–10,000		
	Against MyosinIIA	–	–	–	–	1–5000		
	Against GAPDH	–	–	–	–	1– 2500		
Anti-mouse HRP	Goat anti-Mouse polyclonal antibody						32230	Thermo Fisher Scientific
	Against N-Cadherin	–	–	–	–	1–500		
F-actin	Phalloidin-Rhodamine	–	1–200	1–100	1–200	–	R415	Thermo Fisher Scientific
	Phalloidin-Alexa Fluor 488	–	1–200	1–100	1–200	–	A12379	Thermo Fisher Scientific
	Phalloidin-Alexa Fluor 633	–	–	–	1–50		A22284	Thermo Fisher Scientific
DNA	DAPI (4′,6-Diamidino-2-Phenylindole, Dihydrochloride)	–	1–400	1–400	1–400	–	D1306	Thermo Fisher Scientific

### Whole-mount staining of tumor tissue

Fixed tissue was sliced to 250 µm thick slices in a vibratome (Leica VT1200S) as described before^10,41^. Staining was performed as described above with minor modifications: permeabilization was done with 1% Triton X-100/PBS (v/v) for 1 h at RT (500 µl per tube), all antibody solutions and washing steps were performed using 0.2% Triton X-100/PBS (v/v), under mild shaking conditions (1 mL per tube) and concentration of antibodies was increased (see Table 1, in 150 µl per tube). Incubations with antibodies were done without agitation.

### Confocal imaging

Stained cryosections and whole-mount tissue, as well as stained in vitro cell cultures were imaged using an inverted confocal microscope (Zeiss LSM880NLO) using laser lines 405, 488, 561 and 633 nm and the following objectives: 25×/0.8 OIL, W, Gly LD LCI PL APO (UV) VIS-IR, 40×/1.30 OIL DICII PL APO (UV) VIS-IR and 63×/1.4 OIL DICII PL APO. Images were processed using ImageJ.

Second harmonic imaging of non-stained whole-mount tissue sections was performed in an inverted AOBS two-photon laser-scanning confocal microscope (Leica SP8), coupled with a femtosecond laser (Chameleon Vision II, Coherent Inc.) using a 40×/1.10 HC PL APO CS2 water immersion objective. The excitation wavelength was set at 947 nm and signals were acquired using 3 non-descanned HyD detectors: 525/40 nm (for GFP), 585/40 nm (for tdTomato) and <492 nm (for SHG). The acquisition was performed in resonant mode, with a Z-step of 5 µm and tiling arrays were performed to image large tissue areas. Image stitching was performed using the LAS X software (Leica).

### Segmentation of SHG images to quantify tumor compartmentalization

Tumor slices are generally tilted and folded, hindering the selection of one single plane of the Z-stack to perform 2D image analysis. Conventional maximum intensity or mean intensity projections fail at preserving low intensity regions, which are important to define the areas devoid of stroma. To overcome this, we performed Z-projections based on the intensity of big areas, not single pixels, to preserve the low intensity pixels. To achieve this, we calculated the local most in-focus plane (defined as the plane with maximum standard deviation) along the image using a scanning window of 300 × 300 pixels with an overlap of 0.33. This protocol provided a matrix with the number of the best focus plane in each of these 300 × 300 regions. We resized this matrix to the size of the original image and smoothened it by applying a Gaussian blur (600 pixels radius). This final matrix of Z-planes was used to perform projections of the Z-stacks and build 2D images.

To quantify areas devoid of stroma, we first applied a Gaussian blur (3 pixels radius) to the projected SHG images and applied a user-defined threshold to obtain a binary image labeling all the pixels lacking SHG signal (stromal devoid). We dilated and eroded (3 pixels) this image to fuse adjacent objects and indexed each independent object in the field, which was then filled and smoothened. A stroma-devoided region was defined as any object bigger than 15,565 µm^2^ (circle of ~70 µm radius).

### Laser ablations

CAFs-cancer cells cocultures were imaged using a two-photon laser-scanning microscope (Zeiss LSM880NLO) in single photon mode, using a 40×/1.30 OIL DICII PL APO (UV) VIS-IR objective, and laser lanes 488 and 561. CAF rings were ablated using a Ti:Sapphire laser (Mai Tai DeepSee, Spectra Physics) set at 800 nm and laser power of 10% Image acquisition was started 10 s before the ablation, every 2 s and for a total time of 50 s.

To perform laser ablations in vivo, living tumor tissue slices from control or myosinIIA KO mice were prepared as described previously^10,40^, and placed into a 35 mm glass-bottom culture dish. A slice anchor (SHD-26GH/10; Harvard Apparatus) was placed on top of the tissue slices to minimize sample drift and covered with a drop (100 µl) of DMEM-GlutaMAX (Glibco), supplemented with 1% (v/v) antibiotic-antimycotic (Gibco), 2.5% (v/v) fetal bovine serum, 1% (v/v) Insulin-Transferrin-Selenium (ITS, ThermoFisher Scientific) and 10 ng/mL EGF (Peprotech). The ablation setup was adapted from the in vitro experiments, with laser power set at 20%. Image acquisition was started 10 s before the ablation, every 2 s and for a total time of 50 s. Para-Nitroblebbistatin (50 µM) (Optopharma) was added to 4–5 slices per mouse, incubated at 37 °C, 5% CO_2_ for 2.5 h, and then ablations were performed as described.

### Quantification of tissue recoil

To quantify tissue recoil upon laser ablation, we measured tissue displacements using custom-made PIV with a window size of 16 × 16 pixels and an overlap of 0.75. For each timepoint, we computed tissue displacements relative to the timepoint immediately before ablation.

We measured displacements of mTomato and GFP channels independently. Displacements of each channel were combined according to the segmentation of different cell populations. For all conditions, cancer cell regions (GFP) were manually segmented. For in vitro and in vivo control tumors, this segmentation was enough, since CAFs/stroma express mTomato. For myosin-IIA KO tumors where the stroma has a heterogeneous labeling, threshold-based automatic segmentation was performed to discriminate between KO CAFs (GFP+, mTomato) and the rest of the stroma (GFP−, mTomato+). Based on this segmentation, we build a combined displacement map containing, at each pixel, the displacements of the relevant channel.

To quantify the recoil of cancer cells and CAFs near the cut, we define different regions of interest (ROIs) where displacements will be averaged. We automatically define these regions by dilating the mask of the ablated region 48 pixels for cancer cells and 75 pixels for CAFs, and then finding the overlap between these dilated regions and the cancer cell or the stromal area, respectively. Control ROIs far from the cut were defined by automatically creating ROIs within the cancer cells as far as possible from the cut. We then averaged displacements in each of these ROIs and computed the magnitude and direction of the mean displacement. For ablations parallel to the cancer cell boundary where the cut is not a straight line, we decomposed displacements in perpendicular and tangential components with respect to the cut (as explained above for traction forces) before averaging. We used the mean perpendicular and tangential components to calculate the angle with respect to the cut defined over 360° (−90° towards the cut, 0 or 180° as parallel to the cut, and 90° away from the cut). For laser ablations perpendicular to the cancer cell boundary, the direction of the cut was defined by fitting a straight line to the cut. The angle difference between the mean displacements and the cut was then calculated (−90° towards the cut, 0 or 180° as parallel to the cut, and 90° away from the cut for CAFs and 0° towards the cut, +90 or −90° as parallel to the cut, and 180° away from the cut for the cancer cells).

### Patient-derived xenografts (PDX)

Tumor tissue was obtained from rectal cancer patients at the moment of surgery after chemo-radiotherapy treatment at Institut Curie Hospital, Paris, with the patients’ written consent and approval of the local ethics committee. Samples were collected in DMEM with 10 mmol/L HEPES, 4.5 g/L glucose, 1 mmol/L pyruvate sodium, 200 U/mL penicillin, 200 μg/mL streptomycin, 5 μg/mL ciprofloxacin, 20 μg/mL metronidazole and 2.5 μg/mL fungizone. Small fragments (∼50 mm^3^) were subcutaneously engrafted into the scapular area of anesthetized 5-6 weeks female Nude mice (Crl:NU(Ico)-Foxn1^nu^) (either under xylazine/ketamine or isoflurane anesthesia). Tumor growth was monitored weekly, and tumors were excised when reached a volume between 800 to 1500 mm^3^ and passage into a new recipient mouse (the maximal tumor size approved by the ethical committee was 2000 mm^3^). PDXs were considered as established after 3 consecutive passages.

### Primary CAF lines generation and characterization

Primary mouse CAFs were obtained either from PDX tumor tissue or from tumors generated in SMA-CreER^T2^; R26^mT/mG^; myosin IIA^fl/fl^ mice. For this, mice were sacrificed, and tumors were excised and collected in DMEM Glutamax with 2% (v/v) Antibiotic-Antimycotic (Gibco), 5 μg/mL ciprofloxacin and 20 μg/mL metronidazole, on ice. The tissue was sliced into small pieces of less than 1mm^2^ and carefully plated on top of an 11 kPa polyacrylamide gel coated with 100 µg/ml of collagen I (see gel preparation protocol). Tissue pieces were then cultured in DMEM GlutaMAX supplemented with 10% (v/v) FBS, 1% (v/v) Insulin-Transferrin-Selenium (ThermoFisher Scientific), 2% (v/v) Antibiotic-Antimycotic (Gibco), and Metronidazol/Ciprofloxacin (20 and 5 μg/mL, respectively). The medium was changed every 3 days. Once emerged from the tissue, and while still being in the PAA gels, CAFs were immortalized by retroviral infection of SV40 large T-antigen as described^42^. pBABE-puro SV40 LT was a gift from Thomas Roberts (Addgene plasmid # 13970; http://n2t.net/addgene:13970; EEID: Addgene_13970)^43^. After immortalization, cells were cultured in DMEM GlutaMAX supplemented with 10% FBS and 1% Insulin-Transferrin-Selenium (ThermoFisher Scientific).

Immortalized CAF cultures were characterized for the expression of αSMA, by immunofluorescence and flow cytometry, where CD45, EpCAM and CD31 were used as markers to exclude the presence of immune, epithelial or endothelial cells, respectively. See Table 1 for antibody information and dilutions.

In vitro recombination of CAFs coming from SMA-CreER^T2^; R26^mT/mG^; myosin IIA^fl/fl^ mice was induced using 2 µM 4-hydroxytamoxifen (Sigma Aldrich), for 2 days.

### Quantification of proliferation in CAFs and cancer cell’s cultures

5 × 10^3^ CAFs from all conditions were seeded per well in a 96-well plate, at least in triplicates. Proliferation/viability was then assessed at 24 and 48 h using alamarBlue cell viability reagent (ThermoFisher Scientific). At each timepoint, cells were incubated for 3 h with a 1:10 alamarBlue solution (diluted in complete medium), and then fluorescence was measured in a spectrophotometer using 580/590 nm (excitation/emission) filter settings. To evaluate the effect of CAFs conditioned medium in cancer cell growth, WT and MyosinIIA KO CAFs were grown in 100 cm^2^ plates until reaching 70% confluency. Then, medium was replaced, conditioned for 48 h and added to cancer cells seeded in 96-well plates in triplicates for each condition. Cell growth was then measured using AlamarBlue as described before, 24 and 48 h later.

### Establishment of human primary cancer cell lines and characterization

Primary human cancer cells were obtained from PDX tumors. For this, mice were sacrificed, and tumors were excised and collected in DMEM GlutaMAX with antibiotics (same as above) on ice. Tumors were cut into 0.5 cm^3^ blocks and crushed with a striated plunger from a disposable syringe. The resulting pieces were transferred to a 25 cm^2^ culture flask and incubated in DMEM GlutaMAX, 10% (v/v) FBS, with the same antibiotics at 37 °C, 5% CO_2_. Antibiotics were removed after passage 2. If necessary, controlled trypsinizations were done to remove contaminating fibroblasts. Cancer cells were routinely passed once a week, and the medium was changed twice in between.

Primary cancer cell cultures were characterized by the expression of EpCAM by flow cytometry. CD45, Vimentin and CD31 were used to exclude the presence of immune, fibroblasts or endothelial cells, respectively.

### siRNA

For protein (fibronectin and N-cadherin) depletions using siRNA, CAFs were cultured in standard conditions and transfected using Lipofectamine 3000 (ThermoFisher Scientific). 50×10^4^ CAFs were plated overnight in 6-well plates and then subjected to transfection using 125 nM siRNA. Cells were used 48 h after transfection. For cancer cells-CAFs co-cultures experiments, a second transfection round was performed after co-culture establishment, right before image acquisition, to maximize knockdown efficiency. The specific siRNAs used were: Mouse Cdh2: SI00168252 and SI02666433 (Qiagen), Mouse Fn1: J-043446-09-0002 and J-043446-10-0002 (Horizon Discovery, Perkin Elmer), Negative control: All Stars Negative Control siRNA (1027280, Qiagen).

### Western blot

Cells were scrapped and lysed in RIPA buffer (10 nM Tris HCl pH 7.4, 100 mM NaCl, 1 mM EDTA, 1 mM EGTA, 1% (v/v) Triton X100, 10% (v/v) glycerol, 0.1% (v/v) SDS) with a 1% (v/v) cocktail of protease inhibitors (Sigma-Aldrich). Protein lysates were resolved on 4-20% or 7,5% TGX gels (Miniprotean TGX, BioRad), transferred onto nitrocellulose membranes (Blot Turbo, BioRad) and immunoblotted with the indicated antibodies (Table 1) overnight at 4 °C in 5% (w/v) non-fat milk in PBS-Tween (0.1% v/v), and detected using peroxidase-conjugated secondary antibodies (Table 1), incubated for 1 h at RT in 5% (w/v) non-fat milk in PBS-Tween (0.1% v/v). Signal was revealed using an ECL substrate (Thermo Pierce ECL 2) and visualized using X-ray films.

### Fabrication of polyacrylamide gel substrates

Glass-bottom dishes (World Precision Instruments) were treated with silane (3-(Trimethoxysilyl) propyl methacrylate, Sigma-Aldrich) diluted 1:3 in PBS, for 15 min, rinsed 3 times (5 min each time) with water, and dried out. They were then incubated with a solution of 0.5% Glutaraldehyde/PBS (w/v) for 30 min, rinsed 3 times with water and dried out. Polyacrylamide (PAA) gels of 0.2 or 11kPa (Young modulus) were produced as described previously^44^. Briefly, for 11 kPa, a solution containing 7.5% (v/v) acrylamide, 0.1% (v/v) bis-acrylamide, 0.5% (w/v) ammonium persulphate, 0.05% (w/v) tetramethylethylenediamine and 2% (v/v) of 200-nm-diameter red/green/DAPI fluorescent carboxylate-modified beads was prepared. For 0.2 kPa, the solution contained 3% (v/v) acrylamide, 0.03% (v/v) bis-acrylamide, 0.5% (w/v) ammonium persulphate, 0.05% (w/v) tetramethylethylenediamine and 2% (v/v) of 200-nm-diameter red/green/DAPI fluorescent carboxylate-modified beads. For both stiffnesses, the mix was allowed to polymerize for 1 h on top of the silanized glass, covered with a non silanized 18-mm-diameter coverslip. Beads were not added for gels used to generate primary CAF cultures. After 1 h, the coverslip was removed and the PAA gel surface was incubated with a solution of 2 mg/mL Sulpho-SANPAH under a 265 nm UV light for 10 min. After that, gels were washed once with 10 mM HEPES for 3 min under agitation, and twice with PBS, to remove the excess of Sulpho-SANPAH. Gels were then coated with a solution of 100 or 1 µg/mL (depending on the experiment) of rat-tail Collagen I (Corning) in PBS overnight at 4 °C.

### Fabrication of PDMS stencils

Polydimethylsiloxane (PDMS) membranes were fabricated as explained previously^44^. Briefly, SU8-50 masters containing an array of 150 μm radius circles were raised using conventional photolithography. Uncured PDMS was spin-coated on top of the masters to a thickness lower than the SU8 features (35 μm) and cured at 80 °C for 2 h. A thick border of PDMS was added for handling purposes. Finally, PDMS stencils were peeled off and stored in 96% ethanol until use.

### Cell patterning on PAA gels

PDMS stencils were treated with a solution of 2% Pluronic acid F127/PBS (w/v) (Sigma Aldrich) for one hour. They were then rinsed twice in PBS, let dry for 20 min and carefully placed on top of a well-dried, collagen-coated PAA gel. ~500.000 cancer cells were seeded in a 100 µl drop on top of the PDMS stencil. After 2 h, non-attached cells were removed, and 2 mL of medium supplemented with 10% (v/v) FBS, 2% (v/v) Antibiotic-Antimycotic (Gibco), and Metronidazole/Ciprofloxacin (20 and 5 μg/mL, respectively) was added. The attached cancer cells were then allowed to spread overnight. PDMS stencils were then carefully removed and a cell culture medium containing the same antibiotics was added.

If necessary, cancer cells were labeled at this step. For this, the culture medium was removed, and cells were washed twice with PBS and incubated with a solution of CellTracker Orange (ThermoFisher Scientific) (1:1000 in Hanks’ Balanced Salt Solution, HBSS, Gibco), for 30 min at 37 °C, 5% CO_2_. Cells were then washed three times (once with HBSS and twice with cell culture medium), for 10 min each. If necessary, CAFs were as well stained, before being detached from the culture plate. For this, CAF cultures were incubated with a solution of CellTracker Green (ThermoFisher Scientific) (1:2000 in culture medium without FBS) for 30 min at 37 °C, 5% CO_2_. After staining, CAFs were washed 3 times (10 min each) with a complete culture medium.

To generate cancer cells-CAFs cocultures, the culture medium was removed from the plate containing patterned cancer cell cultures, and a solution containing ~2.5 × 10^5^ CAFs in a 75 µl drop was added on top of the PAA gel surface. Cells were then incubated for 30–60 min to allow CAFs to attach to the gel. Non-attached cells were rinsed out with cell culture medium, and 3 mL of medium supplemented with antibiotics (same as above) were added. Cocultures were incubated at 37 °C, 5% CO_2_, or immediately used for experiments.

### Traction force microscopy

Time-lapse images were acquired on an automatic inverted microscope (Nikon Eclipse Ti-E) equipped with thermal, CO_2_, and humidity control, using a 10X objective (NA 0.5 DIC, air) and controlled through MetaMorph (Universal Imaging). 10–20 cancer cell islands per experimental condition were imaged every 30 or 60 min using a motorized stage. For single-cell traction experiments, cells were imaged using a 20× objective (NA 0.75 DIC, air) for a single time point.

Traction forces were computed using Fourier-transform traction microscopy with finite gel thickness from a gel displacement field as previously described^45^. Gel displacements were obtained using a custom-made particle image velocimetry (PIV). In brief, the fluorescent beads in any experimental time point were compared to a reference image obtained after cell trypsinization at the end of the experiment.

Given the non-normal distribution of traction forces, strain energy instead of average tractions was computed when cell areas between conditions were significantly different. Strain energy was computed by multiplying the traction stress of each pixel by the pixel area and its displacement. The noise was calculated in cell-free areas of the same area and substracted.

### Kymographs and averaging

To perform radial averages of traction forces, cancer cell clusters were segmented at every timepoint (either manually or automatically if cancer cells were fluorescently labeled). We then calculated the shortest (signed) distance of each pixel of the image to the cluster edge. Furthermore, we calculated the normal direction with respect to the cluster edge as described previously^45^ to decompose tractions in radial and tangential components. For every time point, we averaged each of these components according to their distance to the cluster edge to build spatiotemporal kymographs. The discretization for averaging was 1 traction pixel (10.32 × 10.32 μm).

Different cancer cell clusters exhibit slightly different sizes due to experimental variability, hindering the averaging of traction profiles. To avoid averaging artifacts, each traction profile was linearly resized to the average cluster radius of the experimental condition before averaging.

### EdU incorporation assays and in vitro immunostaining

After the specified culture/coculture times, cancer cell/CAFs were incubated for 1 h with a 10 µM EdU solution in a complete medium. Cells were then fixed, and EdU incorporation was revealed as recommended, using the Click-iT EdU Alexa Fluor 647 Imaging kit (ThermoFisher Scientific), and visualized in a confocal microscope.

For immunostainings of in vitro cultures, cells were fixed in 4% paraformaldehyde)/PBS (v/v) for 20 min at RT, permeabilized with 0.2% Triton x100 (Sigma-Aldrich)/PBS (v/v) for 20 min at RT, and blocked with 3% BSA (w/v) in PBS (v/v) solution for 1 h at RT. Primary antibodies were prepared in a 0.05% Tx100/PBS (v/v) solution and incubated overnight in humidified chambers at RT. Cells were then washed 3 times with PBS and incubated with secondary antibodies, DAPI and phalloidin, depending on the staining, for 1 h at RT, washed 3 times in PBS and mounted using AquaPolyMount (Polysciences). Antibodies references and dilutions are listed in Table 1.

### Quantification of YAP localization

YAP localization was assessed by quantifying the 3D correlation between Z-stacks of DAPI and YAP channels of immunostainings. First, cell nuclei were segmented in each plane of the Z-stack using a user-defined threshold. For each plane, the masks of the nuclei were dilated 5 pixels to also include cytoplasmic areas. The rest of the pixels were set to not a number, thus excluding cell-free areas from the analysis. Pearson correlation coefficient between YAP and DAPI images was measured using a 3D scanning window of 32 × 32 × 3 pixels (xyz) with an overlap of 0.75. The window evaluates the correlation along the XY area, thus providing 2D matrices of YAP nuclear correlation. For in vitro images, the window was centered around the plane of maximum intensity in the DAPI channel. For in vivo images, where the tissue is not flat, the scanning window was also allowed to move in Z to center around the plane of maximum local intensity.

For in vitro images, cancer cells were manually segmented to calculate their average YAP nuclear correlation. For in vivo images, which are heterogeneous and contain multiple cell types, cancer cells were automatically segmented using a threshold based on their fluorescence profile: no mTomato and low but positive GFP (to exclude GFP+ CAFs in MyosinIIA KO tumors).

To calculate the nuclear to the cytoplasmic ratio in single cells (Supplementary Fig. 11), the cell nucleus and total area were automatically segmented using a threshold on the DAPI and phalloidin channels, respectively. To avoid measuring YAP intensity in regions of different heights that would affect the values (for example, YAP intensity is always lower at the lamellipodia), the ratios were calculated in two regions in close proximity. To do so, rings of 4 pixels in width where automatically defined at each side of the boundary between the nucleus and the cytoplasm. After subtracting the background, YAP intensity was calculated as the average intensity in each ring.

### Nuclear segmentation and morphometrics

For some quantifications in vivo and in vitro (see below), nuclei were automatically segmented using CellPose in Python 3.9. Before segmentation, a median filter was applied to the images to smoothen the signal. 2D segmentation was performed in the plane of maximum intensity in the DAPI channel. All CellPose masks were imported to Matlab for further analysis.

### Quantification of the evolution in cancer cell number in clusters

To quantify the net increase in cancer cell number in 48 h (Fig. 6a), the same cancer cell clusters were imaged right after seeding the CAFs (0 h) and 48 h after. Cancer cells were incubated with Hoestch (1:5000) for 30 min together with the labeling with Cell Tracker Orange before CAFs were seeded. 2 hours after CAF seeding, cancer cell clusters with and without CAFs were imaged using a spinning disk microscope (Nikon Eclipse Ti-E) equipped with thermal, CO_2_, and humidity control, using a 20× objective (NA 0.45) and a Z-step of 0.5 µm. Images of fluorescent beads embedded in the polyacrylamide gel were taken a fiducial markers. Culture dishes were put back in the cell incubator and fixed after 48 h as explained above. Cell nuclei were stained again with DAPI and the same cancer cell clusters of 0 h were reimaged. Cell nuclei were manually counted using Fiji multi-point tool.

### Quantification of Ki67

Ki67 expression was heterogeneous within the cancer cell cluster, making it difficult to define Ki67+ and Ki67− cells. To overcome this, average intensity in the nuclei of all the cancer cells was averaged.

In vitro, nuclei and cancer cells were automatically segmented using a user-based intensity threshold. Cancer cell mask and nuclei mask were multiplied to exclude CAF nuclei, obtaining a mask of cancer cell nuclei. This mask was used to quantify the average intensity of nuclear Ki67 in the cancer cell cluster. All average intensities were divided by the average intensity of the “Free growth” condition to normalize the measurement.

In vivo, nuclei were automatically segmented using CellPose as explained above. Only nuclei in the cancer cell regions were quantified by segmenting cancer cell areas based on their fluorescence using intensity thresholds (mTomato-, GFP+ but low, to exclude Myosin IIA KO CAFs).

### Quantification of Phospho-histone H3 and EDU^+^ cells

For in vitro staining, cancer cell nuclei and PHH3/EDU^+^ nuclei were manually counted in 3D using the Fiji multi-point tool. To quantify the proportion of PHH3/EDU^+^ cells in each cancer cell cluster, the number of PHH3/EDU^+^ were divided by the total number of cells.

For in vivo staining, cancer cell nuclei were automatically segmented using CellPose as explained above. PHH3/EDU^+^ cancer cells were manually quantified and divided by the total number of cancer cells in the field of view.

### Quantification of Cleaved-caspase 3

Cleaved-caspase 3 (CC3) signal is often diffuse and thus counting the number CC3^+^ cells is extremely challenging. Instead, we quantified the CC3^+^ area divided by the total number of cancer cells in the cluster (in vitro) or by the area covered by cancer cells (in vivo). For this, we segmented the CC3^+^ area using an intensity threshold. Cancer cells were counted manually using the Fiji multi-point tool.

### Pillar microfabrication

The experimental pipeline to generate PAA microfabricated pillars is summarized in the Supplementary Fig. 7. At first, a negative SU8-50 photoresist (MicroChem) is coated onto a silicon wafer surface and patterned by conventional photolithography. In particular, an array of circular wells (50 µm radius, 50 µm deep) was fabricated using a chromium photomask (produced by direct laser lithography, Heidelberg µPG101) and the exposure-masking system UV-KUB2 (Kloé), following the photoresist manufacturer protocol. The well depth and surface roughness were verified by optical profilometer (Veeco). The SU8 master mold was then separated into individual array sub-units using a diamond scribe. To coat the flat surface of the SU8 master, 1 cm^2^ PDMS stamps (~3 mm thick) were washed with 70% ethanol, rinsed with distilled water, and incubated for 1 h with 200 µl of a 100 µg/mL fibronectin solution (Corning). The excess of fibronectin was then aspirated and the PDMS surface was dried out thoroughly. SU8 masters were plasma treated for 1 min at high power (Harrick Plasma). Fibronectin-coated PDMS stamps were then put in contact with the SU8 plasma-treated surface and pressed down for 30 s to allow fibronectin transfer between surfaces. Effective transfer was verified by checking the presence of circular non-transferred areas in the PDMS stamp in an inverted phase contrast microscope.

Once coated with fibronectin, SU8 masters were used to microfabricate PAA pillars. For this, 33 mm bottom-glass dishes (World Precision Instruments) were treated as before (Silane + Glutaraldehyde). PAA gels of 11 kPa (Young modulus) were produced using a solution of 7.5% (v/v) acrylamide, 0.1% (v/v) bis-acrylamide, 0.5% (w/v) ammonium persulphate, 0.05% (w/v) tertamethylethylenediamine, 0.6% (w/v) Acrylic acic N-hydroxysuccinimide ester (stock 10 mg/mL in DMSO) and 2% (v/v) of 200-nm-diameter red fluorescent carboxylate-modified beads, in PBS. A 25 µl drop of this solution was added to the treated glass and the SU8 master was placed on. PAA gels were allowed to polymerize for 1 h at RT. After, 3 ml of PBS were added and incubated for 30 min to facilitate lifting-off of the SU8 master, which was achieved with the help of a scalpel. Gels were then washed once with PBS and incubated with a solution of 100 µg/mL of fibronectin for 1 h at RT to improve cell attachment. Gels were then washed twice with PBS and used immediately or stored at 4 °C for a maximum of 24 h.

### Pillar compression assay

To assess CAF pillar compression, a 100 µl drop containing ~200.000 pre-stained CAFs (CellTracker Green, ThermoFisher Scientific) was added on top of each pillar-containing PAA gel and incubated for 30-60 min at 37 °C, 5% CO_2_ to allow attachment of cells. Non-attached cells were rinsed with culture medium and 3 mL of culture medium supplemented with 10% (v/v) FBS, 1% (v/v) Insulin-Transferrin-Selenium (ThermoFisher Scientific), 2% (v/v) Antibiotic-Antimycotic (Gibco), and Metronidazol/Ciprofloxacin (20 and 5 μg/mL, respectively). Cells were then incubated overnight at 37 °C, 5% CO_2_.

Pillars were imaged on a spinning disk microscope (Nikon Eclipse Ti-E) equipped with thermal, CO_2_, and humidity control, using a 20× objective (NA 0.75 Water) and a Z-step of 0.5 µm. A reference stack was acquired for each position after cell trypsinization at the end of the experiment. In drug perturbation experiments, pillars were initially imaged and then drugs were added on-stage (20 µM Para-Nitroblebbistatin (Optopharma), 50 µM Y27632 (SigmaAldrich) and incubated for 2 h. The same pillars were then re-imaged and a final reference stack was obtained after cells trypsinization.

### Pillar compression analysis

The CAF-induced deformation of each pillar surface was quantified using a custom-made 3D PIV in Matlab^15^. The contact area between CAFs and the pillar was determined as the intersection of their respective fluorescent signals. The 3D traction field applied on the pillar was calculated, from the 3D deformation field, through a Finite Element Method by using ABAQUS (Dassault Systemes), in the large deformation regime. We prescribed the 3D displacement field as the boundary condition for the nodes that are at the contact area between the CAFs and the pillar, and the rest of the boundary was considered traction free, effectively constraining the tractions to the contact region. The pillar was modeled as a Neo-Hookean hyperelastic material. The projection of the tractions in the normal and tangential directions, unwrapping of the traction maps on the surface of the pillar, post-processing and averaging were performed in Matlab.

### RNA sequencing

RNA was extracted from CAFs from all conditions using the RNeasy mini kit (Qiagen), performing a DNase treatment step to remove possible contamination with genomic DNA. RNA quantity was measured in a NanoDrop and RNA integrity was evaluated in a TapeStation (Agilent). All samples had RNA integrity numbers (RIN) higher than 8.5. Preparation of libraries and transcriptome sequencing was performed by Novogene Co., Ltd. (Cambridge, UK) on an Illumina HiSeq 4000 sequencer. Genes with adjusted p-value < 0.01 and |log2(FoldChange)| > 1,−1 were considered as differentially expressed. Volcano and bubble plots were built using SRplot. Gene Ontology enrichment analyses were performed using GeneCodis4^46^.

### Supplementary information

Supplementary Information

Description of Additional Supplementary Files

Supplementary Video 1

Supplementary Video 2

Supplementary Video 3

Supplementary Video 4

Supplementary Video 5

Supplementary Video 6

Supplementary Video 7

Supplementary Video 8

Supplementary Video 9

Supplementary Video 10

Supplementary Video 11

### Source data

Source Data

## Data Availability

All data are available upon request. RNAseq datasets can be accessed through NCBI GEO, accession number: GSE242846. Source data are provided with this paper.
