# Supplementary material for: Cancer-associated fibroblasts actively compress cancer cells and modulate mechanotransduction
Source: Nat Commun. 2023 Nov 1;14:6966. doi: 10.1038/s41467-023-42382-4 (PMC10618488; doi:10.1038/s41467-023-42382-4)
Supplement: Supplementary file 1 — Supplementary Information [file 41467_2023_42382_MOESM1_ESM.pdf]

Supplementary Information

Cancer-associated fibroblasts actively compress cancer cells and modulate mechanotransduction

Jorge Barbazan[1]†, Carlos Pérez-González[1]†, Manuel Gómez-González[2], Mathieu Dedenon[3], Sophie Richon[1], Ernest Latorre[2], Marco Serra[3], Pascale Mariani[4], Stéphanie Descroix[3], Pierre Sens[3], Xavier Trepat[2,5,6,7]*, Danijela Matic Vignjevic[1]*

Correspondence to: danijela.vignjevic@curie.fr, xtrepat@ibecbarcelona.eu

Supplementary figures and captions

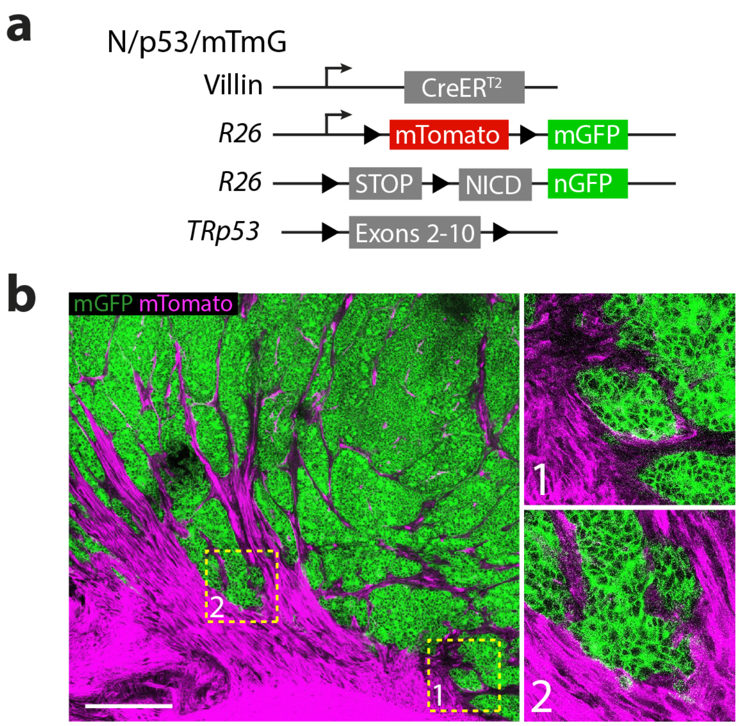

Supplementary Data Figure. 1. CAFs form intratumoral capsules
a, Schematic representation of the NICD/p53/mTmG mouse model. **b,** Representative image of a NICD/p53/mTmG intestinal tumor (from N=3 independent experiments). Cancer cells (membrane-GFP, green), stromal cells (membrane-tdTomato, magenta). Scale bar, 300 µm. Right panels, magnified boxed regions 1 and 2.

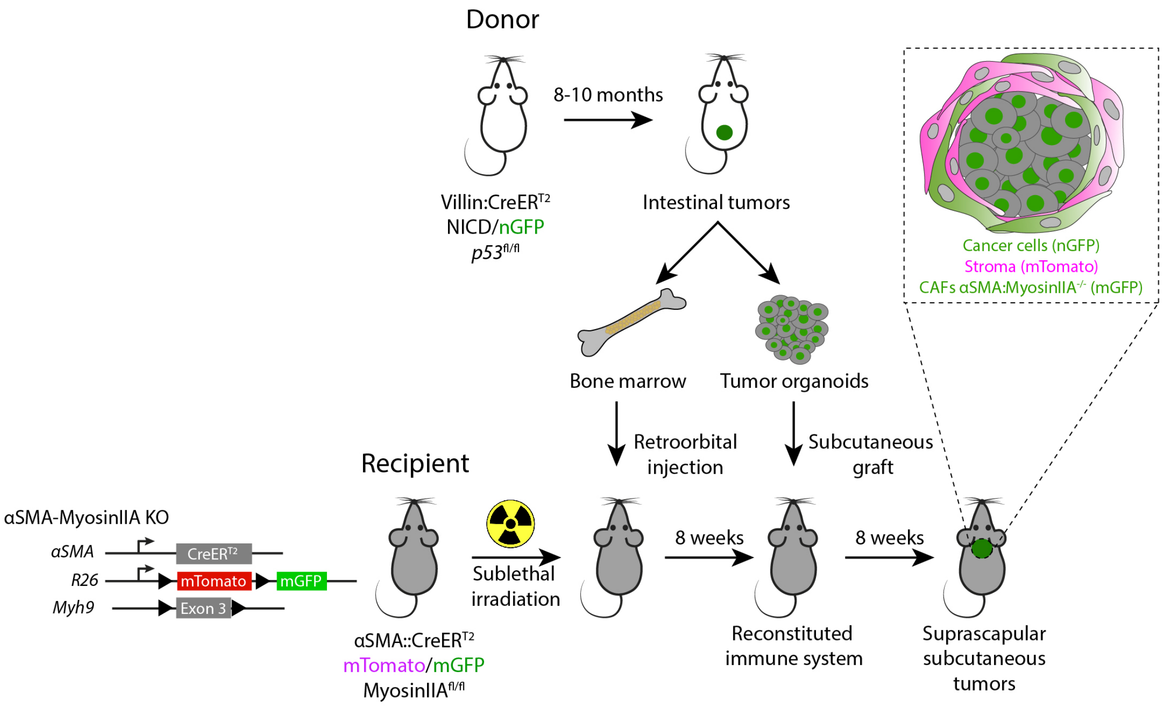

Supplementary Data Figure. 2. Generation of subcutaneous tumors in αSMA-MyosinIIA KO mice

Left, schematic representation of the αSMA-MyosinIIA KO mouse model. Right, experimental design for the engraftment of NICD/p53/mTmG tumor organoids in αSMA-MyosinIIA KO mice.

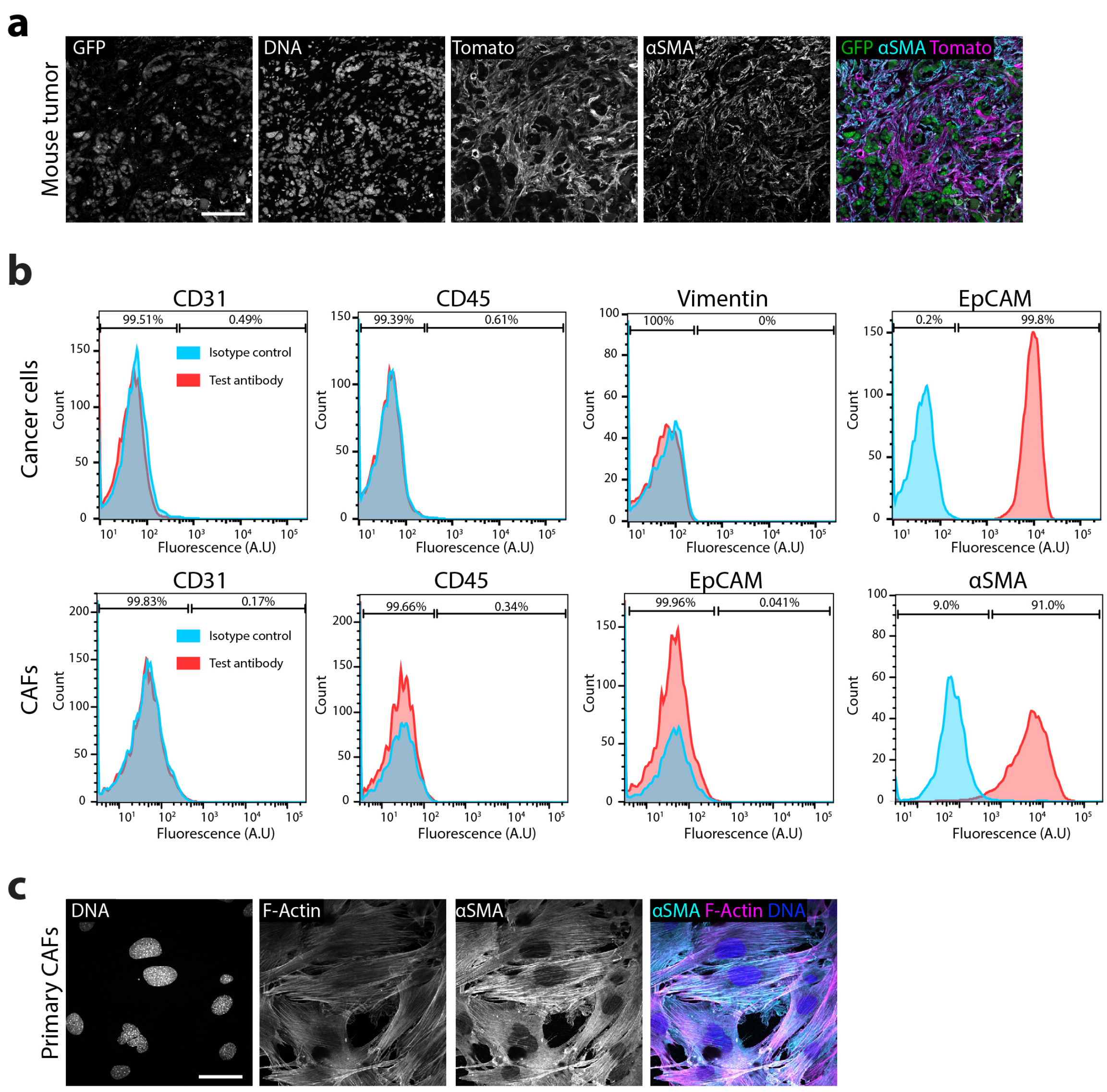

Supplementary Data Figure. 3. Characterization of primary CAFs and cancer cells

a, Representative (from N=5 mice) image of a subcutaneous tumor stained for αSMA (cyan) and DNA (DAPI). Cancer cells express nuclear GFP (green) and the stroma express mTomato (magenta). Scale bar, 100 µm **b**, Flow cytometry analysis of the expression of CD31, CD45, Vimentin and EpCAM in primary cancer cells, and of CD31, CD45, EpCAM and αSMA in primary CAFs. The percentages (%) of stained cells (red) are plotted relative to the basal signal of an isotype control (light blue). **c**, Representative images of primary CAFs stained for F-Actin (phalloidin, magenta), αSMA (cyan) and DNA (DAPI, blue). N=2 independent experiments. Scale bar, 50 µm.

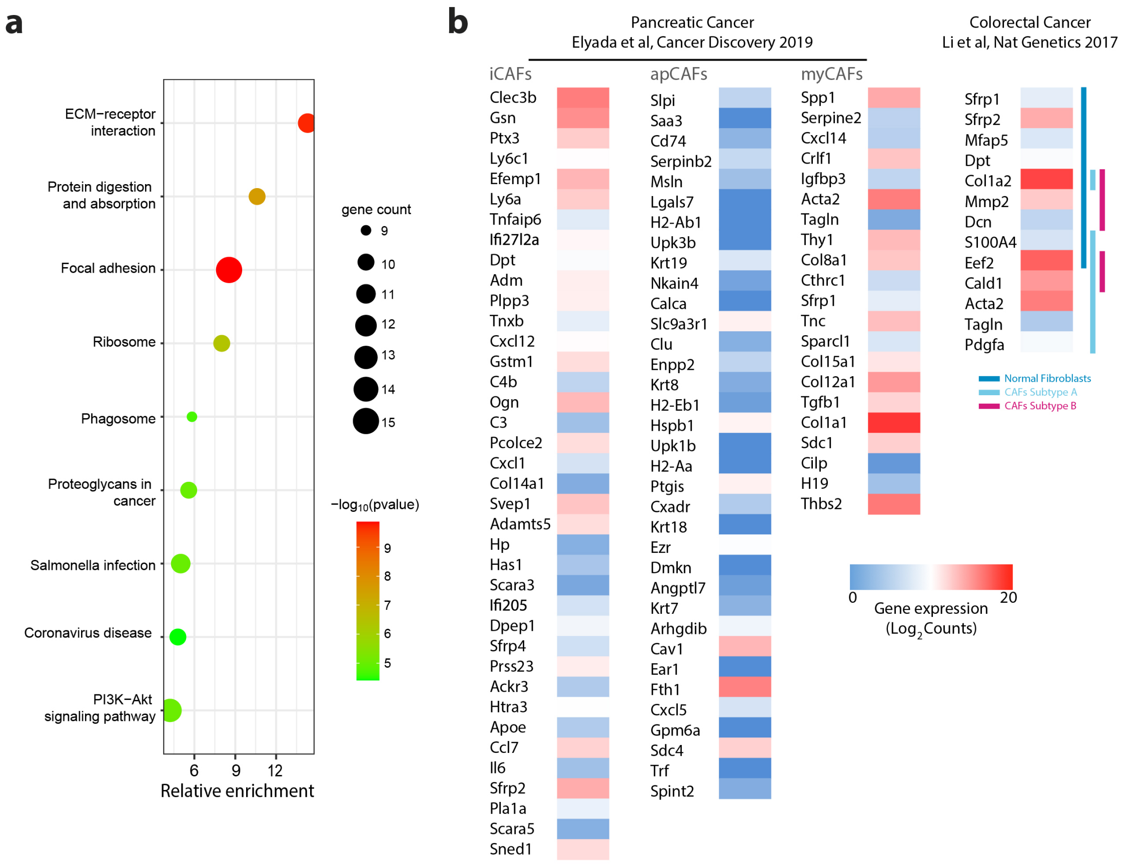

Supplementary Data Figure. 4. Characterization of primary CAFs by RNA sequencing

a, Bubble plot representing the main gene ontology terms (KEGG) enriched when considering the top 100 expressed genes in wild type CAFs. **b,** Expression of selected gene sets in wild type CAFs, previously defined as markers for different CAF subtypes in pancreatic (iCAFs, apCAFs and myCAFs, Eylada et al. 2019) (left), and colorectal cancer (normal fibroblasts, CAFs subtype A and CAFs subtype B, Li *et al.* 2017) (right).

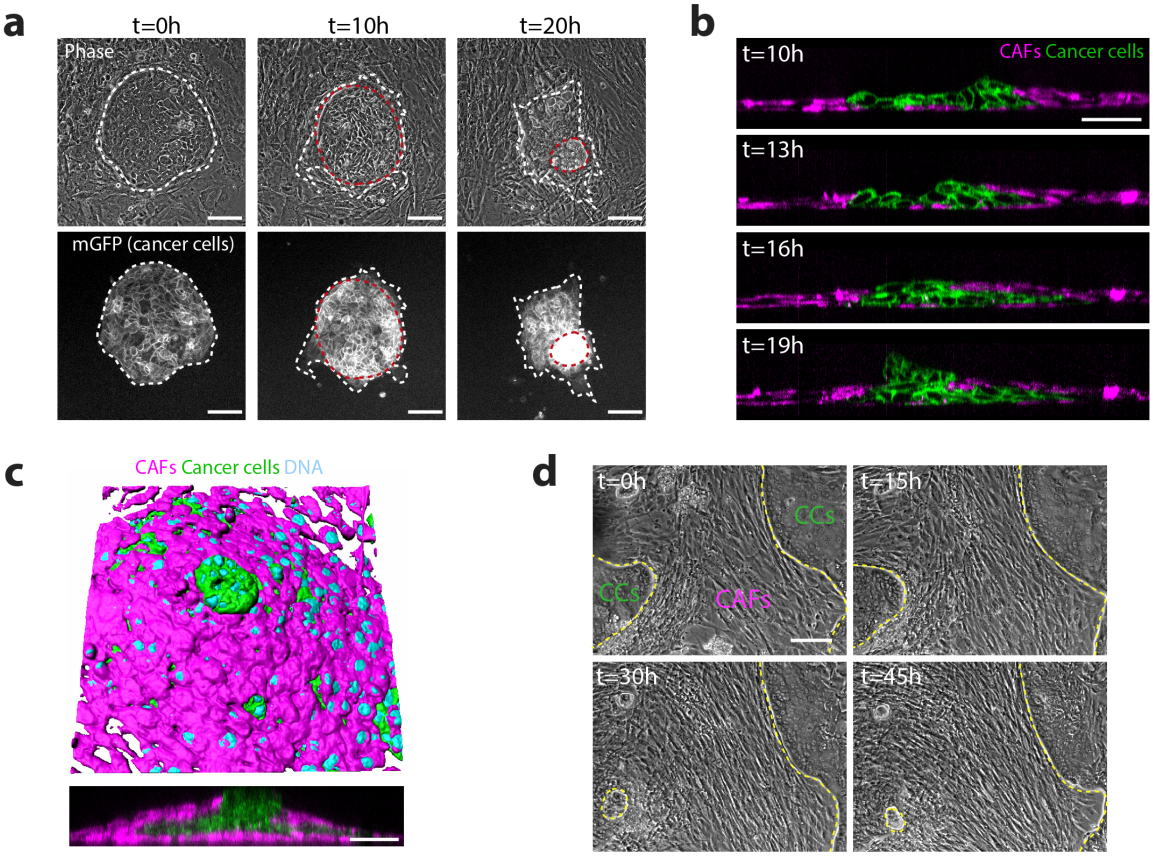

Supplementary Data Figure. 5. CAFs encapsulate and deform N/p53 tumor cell clusters
a, Representative images (from n=68 positions, N=1) of CAFs cocultured with N/p53 tumor cells, evolving over time. Top panels, phase contrast. Lower panels, cancer cells (membrane-GFP, green) Red dashed lines delineate the contour of CAFs ring. White dashed lines delineate the contour of the tumor cell cluster. Scale bar, 100 µm. **b**, Representative (from n=14 positions, N=1) orthogonal images of CAFs cocultured with N/p53 tumor cells, evolving over time. Cancer cells (membrane-GFP, green) and CAFs (CellTracker, magenta). Scale bar, 100 µm. **c**, 3D rendering of CAFs-N/p53 tumor cells co-cultures after 24h in culture (from n=6 positions, N=1). Cancer cells (membrane-GFP, green) and CAFs (CellTracker, magenta), DNA (DAPI, cyan). Lower panel, orthogonal Z section. Scale bar: 50µm. **d**, Representative images (from n=4 positions, N=1) of CAFs and cancer cells that exited a PDX tissue fragment and spontaneously organized over time. Yellow dashed lines represent the CAFs edge and ring. Scale bar, 100 µm.

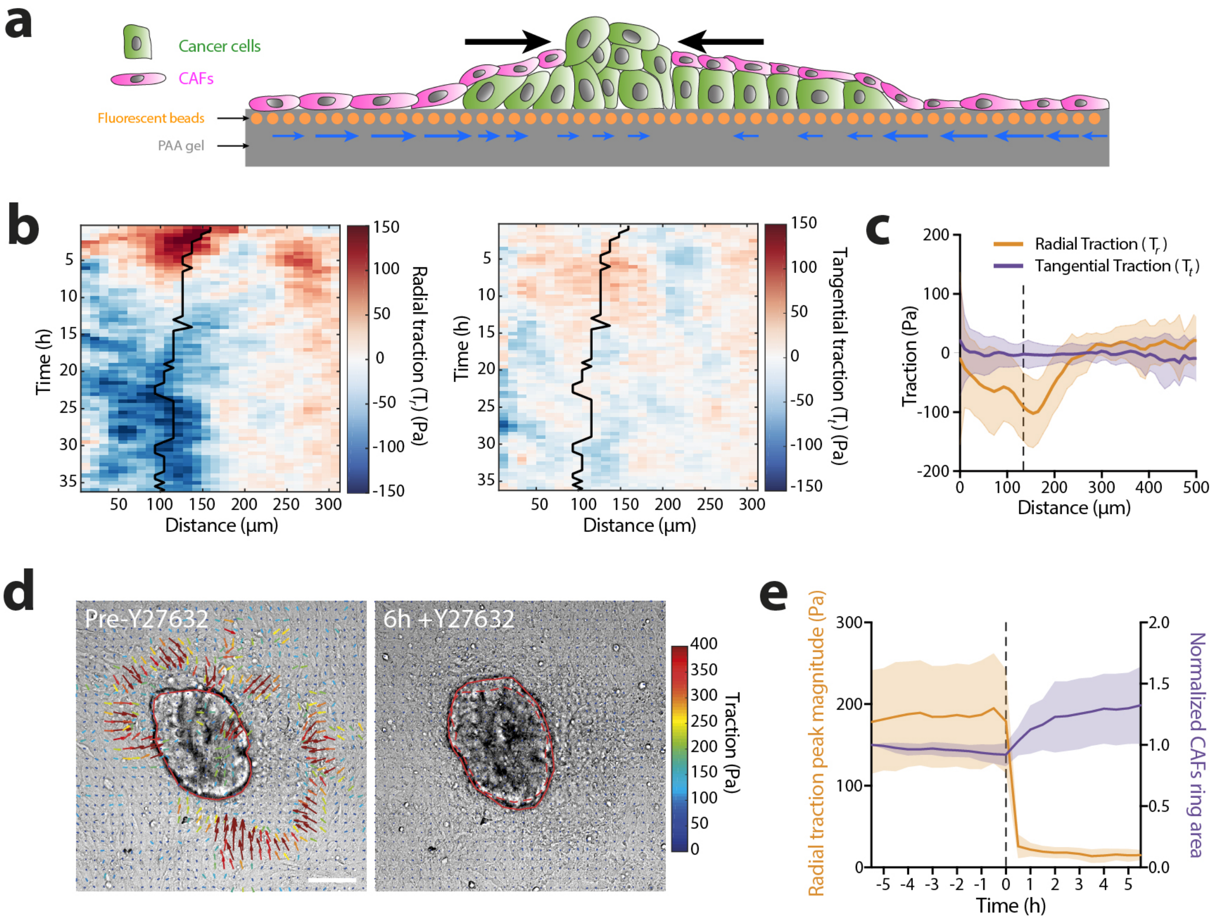

Supplementary Data Figure. 6. CAFs compress cancer cells using actomyosin contractility
a, Schematic representation of the interpretation of force patterns generated by CAFs during cancer cells deformation and budding. Contraction of CAFs rings generate inward pointing forces (black arrows) that are transmitted through the CAFs monolayer to the polyacrylamide (PAA) gel (grey), generating a pattern of inward pointing radial tractions (blue arrows). **b**, Representative kymograph of circumferentially averaged radial (left) and tangential (right) tractions as a function of the distance to the center of the cancer cell cluster. Solid black line represents the cancer cell cluster contour. Outwards pointing radial tractions are defined as positive, and inwards pointing radial tractions are negative. Tangential tractions pointing clockwise are defined as positive, and tangential tractions pointing counterclockwise are negative. **c**, Circumferentially averaged tangential tractions T_t (purple) and radial tractions T_r (orange) as a function of the distance to the cancer cell cluster center after 36h of co-culture. Black dashed line represents the cluster boundary. Data represented as mean ± SD, n=49 clusters from N=3 independent experiments. **d**, Traction maps overlaid on a DIC image of cancer cell-CAFs after ≈40h co-culture (Pre-Y27632) and 6h after addition of Y27632 (6h+Y27632) (quantified in panel e). Solid red line represents the contour of CAFs ring; dashed line represents the contour of CAFs ring before Y27632 addition. Scale bar, 100 µm. **e**, Circumferentially

averaged radial traction peak magnitude near the boundary of the cancer cell cluster (orange) and CAFs ring area (purple) normalized to the initial ring size, as a function of time. Dashed black line represents the moment of Y27632 addition. Data represented as mean ± SD, n=30 clusters from N=3 independent experiments.

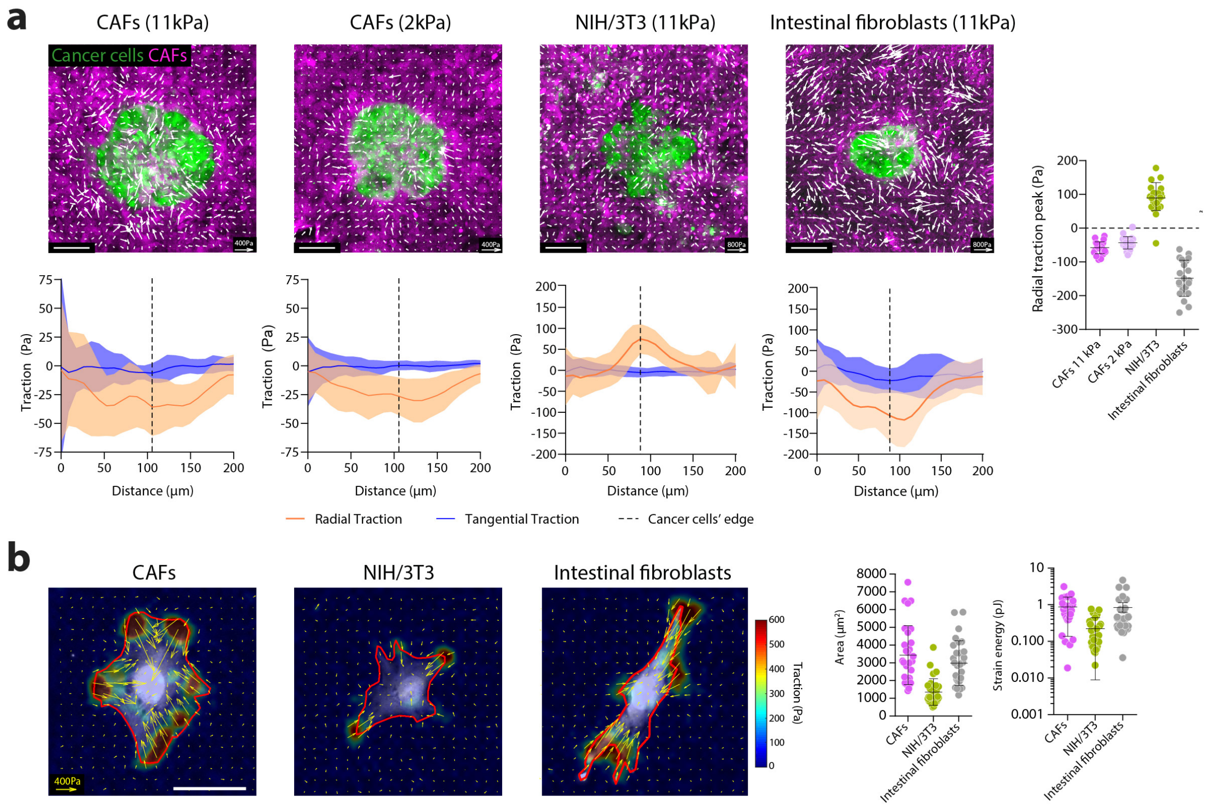

Supplementary Data Figure. 7. Compression assay and single traction forces on normal fibroblasts and CAFs seeded on different stiffnesses.

a, Top: Traction force maps (white vectors) overlaid with fibroblasts (magenta) and cancer cell clusters (green) 48 hours after seeding the fibroblasts. From left to right, fibroblasts are: CAFs control on 11kPa gels, CAFs control on 2kPa gels, NIH/3T3 normal fibroblasts on 11 kPa gels, and mouse normal intestinal fibroblasts on 11kPa. Scale bar 100 µm. Bottom: Radial and tangential traction profiles as a function of the distance to the cluster center for the different conditions. Vertical dashed line indicates the boundary between the cancer cells and the fibroblasts. Outwards pointing radial tractions are defined as positive, and inwards pointing radial tractions are negative. Tangential tractions pointing clockwise are defined as positive, and tangential tractions pointing counterclockwise are negative. Right graph: radial traction peak in each condition, defined as the traction peak in the radial traction profile within a disk of ~50µm around the boundary between the cancer cells and the fibroblasts. Data represented as mean ± SD. n=25 clusters with CAFs 11 kPa, n=25 clusters with CAFs 2 kPa, n=25 clusters with NIH/3T3, and n=21 clusters with intestinal fibroblasts, from N=2 independent experiments. **b**, Representative traction maps overlaid on CellTracker-labelled control CAFs, NIH/3T3 and mouse normal intestinal fibroblasts. Solid red line represents the cell contour. Scale bar, 50 µm. Scale vector, 400 Pa. Right dot plots, quantification of cell area and strain energy. Data

represented as mean ± SD. n= 30 cells from all experimental conditions from N=2 independent experiments.

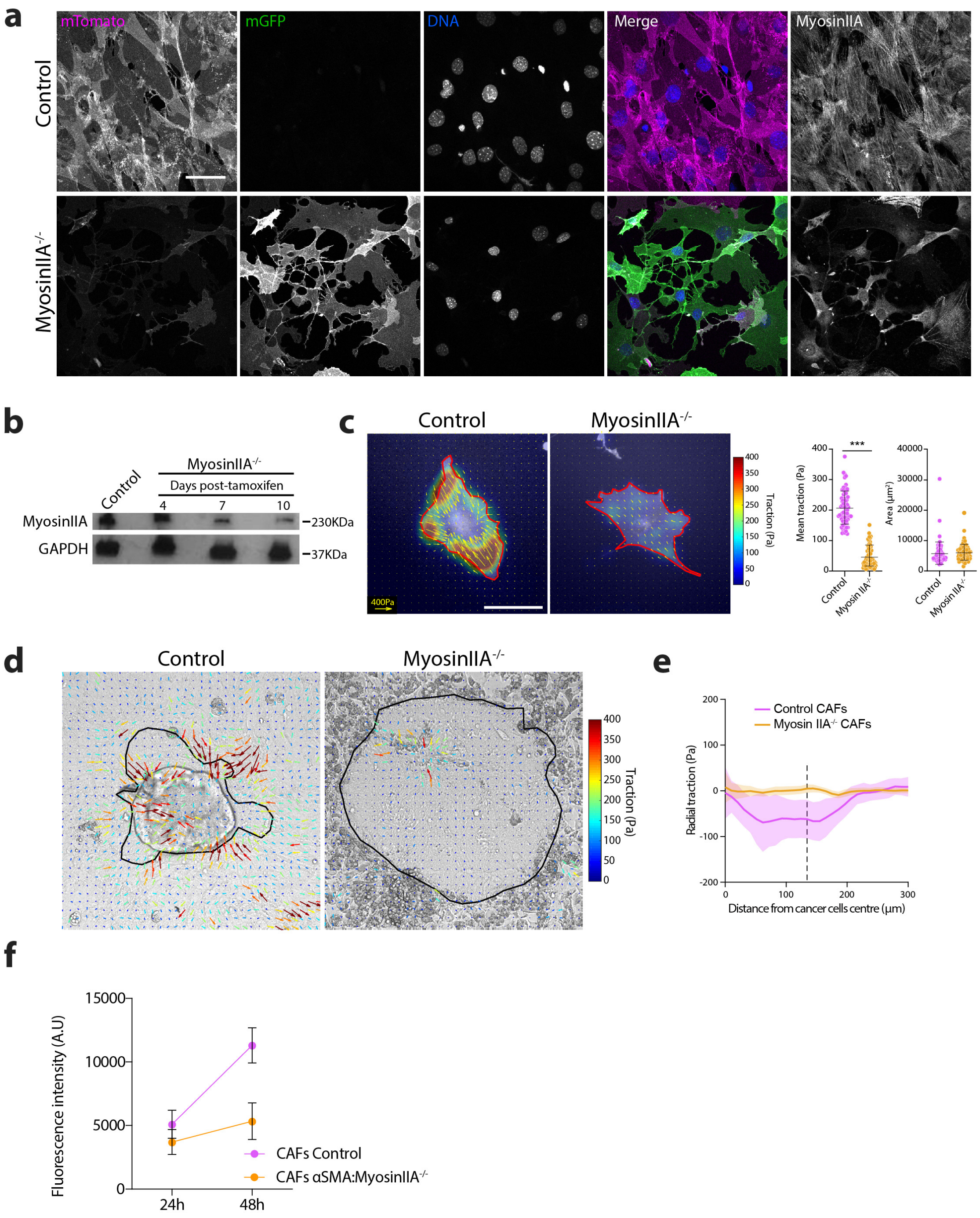

Supplementary Data Figure. 8. MyosinIIA KO CAFs do not exert traction forces or deform cancer cells

a, Representative images (from n=7 images, N=1 independent experiment) of control (upper panels) and MyosinIIA KO (lower panels) CAFs after 7 days of in vitro knockout induction with 4-hydroxytamoxifen. Control CAFs (membrane tdTomato, magenta), KO CAFs (membrane-GFP, green, and low levels of membrane-tdTomato, magenta). Cells were stained for DNA

(DAPI, blue) and MyosinIIA (right panels). Scale bar, 50 µm. **b**, Western blot for MyosinIIA (230KDa) in control and MyosinIIA KO CAFs after 4, 7 and 10 days of knockout induction. GAPDH (37KDa) was used as loading control (N=1). **c**, Representative traction maps (quantified on right dot plots) overlaid on images of control (membrane-tdTomato) and MyosinIIA KO (membrane-GFP) single CAFs after 7 days of in vitro knockout induction. Solid red line represents the cell contour. Scale bar, 50µm. Scale vector, 400 Pa. Right dot plots, quantification of mean traction and area per cell. Data represented as mean ± SD. n=71 cells, from N=3 independent experiments. *** $p<0.001$, Mann Whitney two-tailed test. **d**, Representative traction maps overlaid on a DIC image of a cancer cell and control (left) or myosin IIA KO (right) CAFs after 48h of culture (quantified in panel e). Black line represents the contour of the cancer cell cluster Scale bar, 100 µm. **e**, Circumferentially averaged radial tractions T_r in co-cultures of cancer cells and control (magenta) and MyosinIIA KO (orange) CAFs after 48h, as a function of the distance to the cancer cell cluster center. Black dashed line represents the cluster boundary. Data represented as mean ± SD. Control, n=36 clusters, MyosinIIA KO, n=30 clusters, from N=2 independent experiments. **f,** Fluorescence intensity as a readout of proliferation in control and Myosin IIA KO CAFs from N=2 independent experiments.

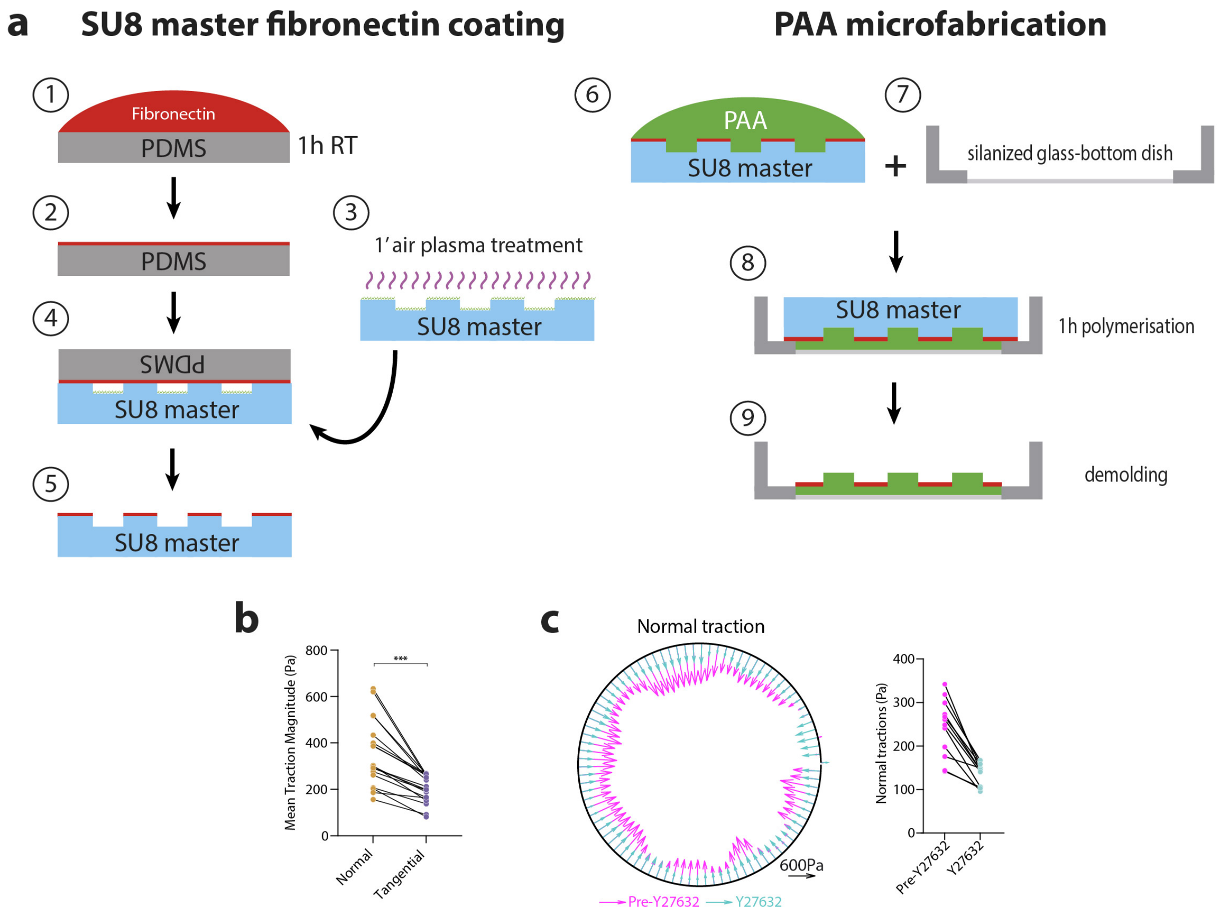

Supplementary Data Figure. 9. Pillar compression depends on CAFs contractility

a, Schematic representation of the methodological approach for pillar microfabrication. **b**, Mean normal and tangential traction magnitude in control pillars. n=18 pillars, from N= 3 independent experiments. *** p<0.001, Wilcoxon matched-pairs signed rank test. **c**, Representation of CAF normal tractions averaged across pillar height, on a representative pillar before (magenta) and after (blue) Y27632 treatment. Scale vector, 600 Pa. Right dot plot, quantification of mean normal traction for each pillar before and after Y27632. n=12 pillars, from N=2 independent experiments.

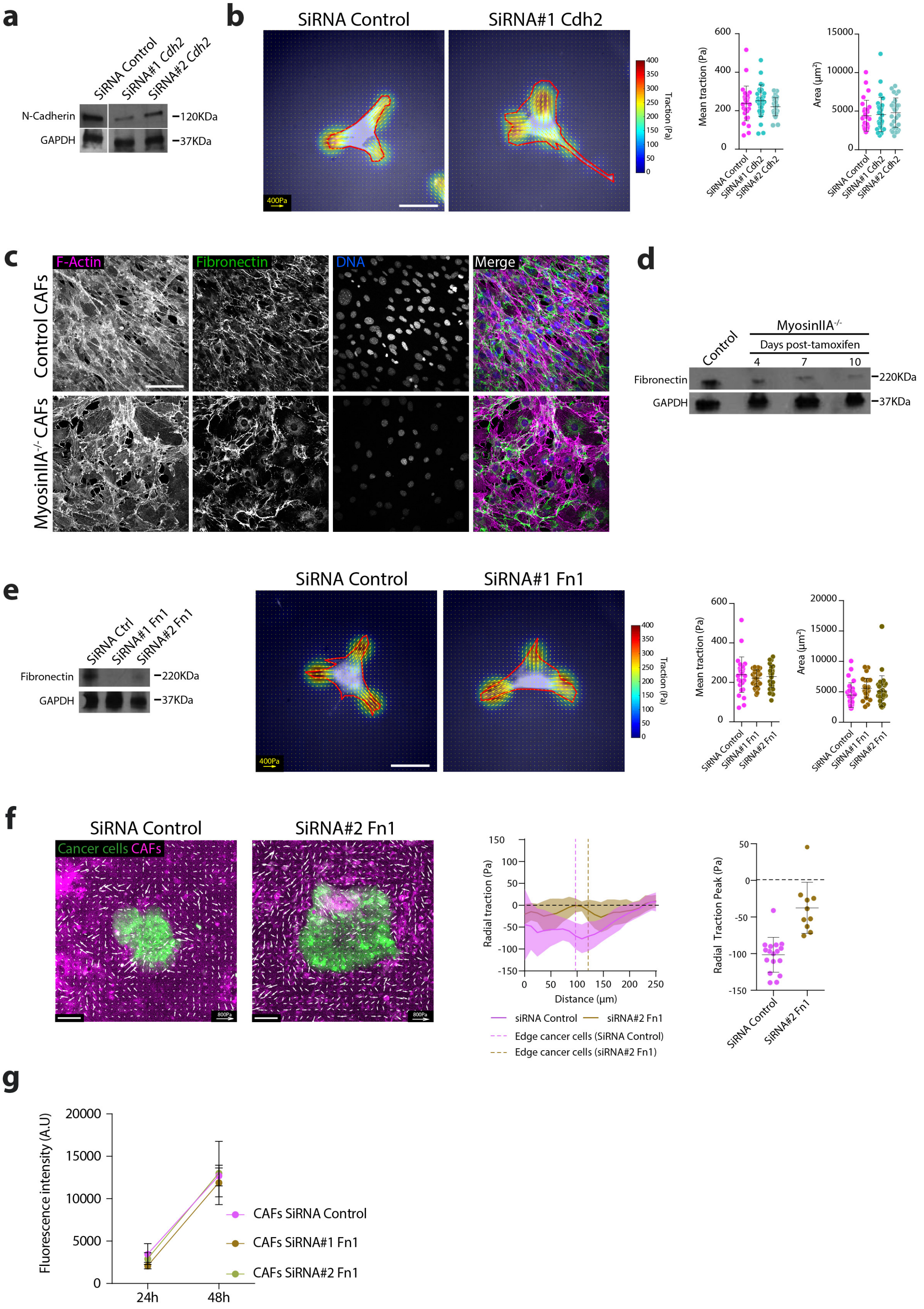
a
SiRNA Control
SiRNA#1 Cdh2
SiRNA#2 Cdh2
N-Cadherin
GAPDH
120KDa
37KDa
b
SiRNA Control
SiRNA#1 Cdh2
Traction (Pa)
400Pa
Mean traction (Pa)
Area (µm²)
c
F-Actin
Fibronectin
DNA
Merge
Control CAFs
MyosinIIA-/- CAFs
d
MyosinIIA-/-
Days post-tamoxifen
Control
4
7
10
Fibronectin
GAPDH
220KDa
37KDa
e
SiRNA Ctrl
SiRNA#1 Fn1
SiRNA#2 Fn1
Fibronectin
GAPDH
220KDa
37KDa
SiRNA Control
SiRNA#1 Fn1
Traction (Pa)
400Pa
Mean traction (Pa)
Area (µm²)
f
SiRNA Control
SiRNA#2 Fn1
Cancer cells CAFs
800Pa
Radial traction (Pa)
Distance (µm)
siRNA Control
siRNA#2 Fn1
Edge cancer cells (SiRNA Control)
Edge cancer cells (siRNA#2 Fn1)
Radial Traction Peak (Pa)
g
Fluorescence intensity (A.U)
24h
48h
CAFs SiRNA Control
CAFs SiRNA#1 Fn1
CAFs SiRNA#2 Fn1

Supplementary Data Figure. 10. Fibronectin and N-cadherin knockdown does not affect force generation at single cell level

a, Western blot for N-Cadherin in control and two independent Cdh2 SiRNA CAFs. GAPDH was used as loading control. N=1. **b**, Representative traction maps (quantified in right dot plot) overlaid on phase contrast images of control and SiRNA#1 Cdh2 single CAFs. Solid red line represents the cell contour. Scale bar, 50 µm. Scale vector, 400 Pa. Right dot plots, quantification of mean traction and area per cell. Data represented as mean ± SD. Control, n=28 cells, SiRNA#1 Cdh2, n=30 cells, SiRNA#2 Cdh2, n=30 cells from N=2 independent experiments. **c**, Representative images of control (upper panels) and MyosinIIA KO (lower panels) CAFs, after 7 days of in vitro knockout induction with 4-hydroxytamoxifen. Cells were stained for F-actin (Phalloidin, magenta), Fibronectin (Green) and DNA (DAPI, blue). Scale bar, 100µm. N=1. **d**, Western blot for Fibronectin in control and MyosinIIA KO CAFs after 4, 7 and 10 days of knockout induction. GAPDH was used as loading control. N=1. **e**, Left: Western blot for Fibronectin in control and SiRNA Fibronectin CAFs. GAPDH was used as loading control. N=1. Right: Representative traction maps overlaid on phase contrast images of control and fibronectin-KD CAFs (SiRNA#1). Solid red line represents the cell contour. Scale bar, 50 µm. Scale vector, 400 Pa. Right dot plots, quantification of mean traction and area per cell. Data represented as mean ± SD. Control, n=28 cells, SiRNA#1 Fn1, n=29 cells, SiRNA#2 Fn1, n=30 cells, from N=2 independent experiments. **f,** Traction maps (quantified in right plots) overlaid on a fluorescent image of CAFs (magenta) and cancer cells (green) 48 hours after seeding. CAFs were transfected with siRNA control (left) or siRNA#2 Fn1 (right). Graph: average radial traction profiles (left) and radial traction peaks (right) of CAFs siRNA control and siRNA#2 Fn1. Vertical dashed lines indicate the boundary between the cancer cells and the CAFs. Data are presented as mean ± SD. n=17 clusters for siRNA control and n=10 clusters for siRNA#2 fibronectin, from N=2 independent experiments. **g,** Fluorescence intensity as a readout of proliferation in CAFs siRNA control, siRNA#1 Fn1 and siRNA#2 Fn1, from N=2 independent experiments.

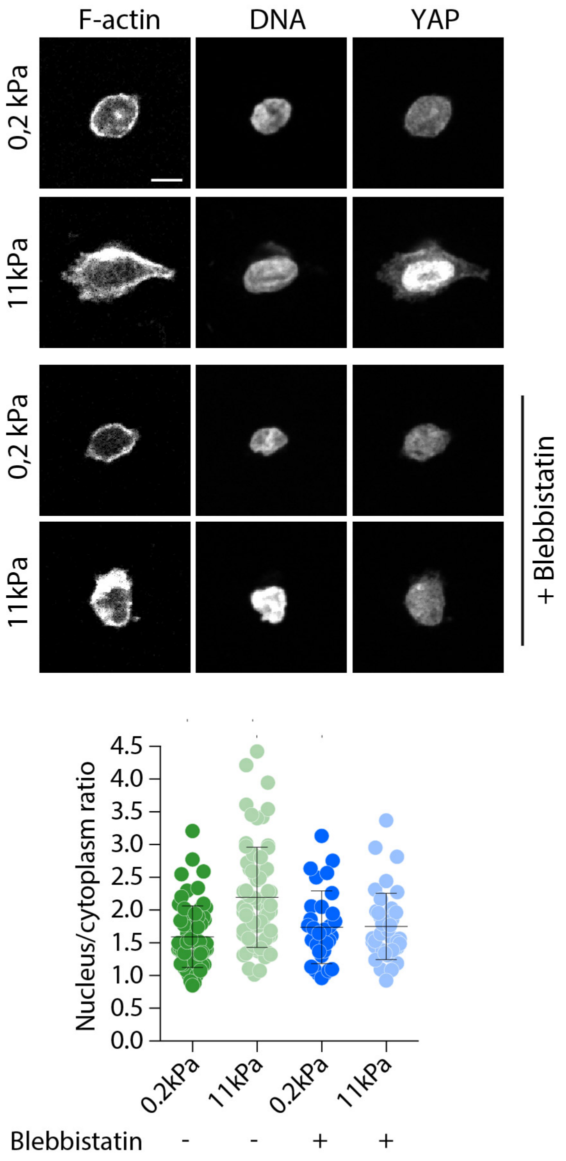

Supplementary Data Figure. 11. Cancer cells mechanosense substrate stiffness through YAP. Upper panel: staining of YAP, F-Actin and DNA in single cancer cells seeded in substrates of different rigidities (0,2 and 11 kPa), coated with 1µg/mL of collagen 1, and treated or not with blebbistatin. Scale bar: 10µm. Lower panel: quantification of YAP nucleus/cytoplasm ratio per cell. Data represented as mean ± SD. n= 68 cells for 0,2 kPa, n=71 cells for 11 kPa conditions from N=3 independent experiments. n= 32 cells for 0,2 kPa + Blebbistatin, n= 42 cells for 11 kPa + Blebbistatin, from N=2 independent experiments.

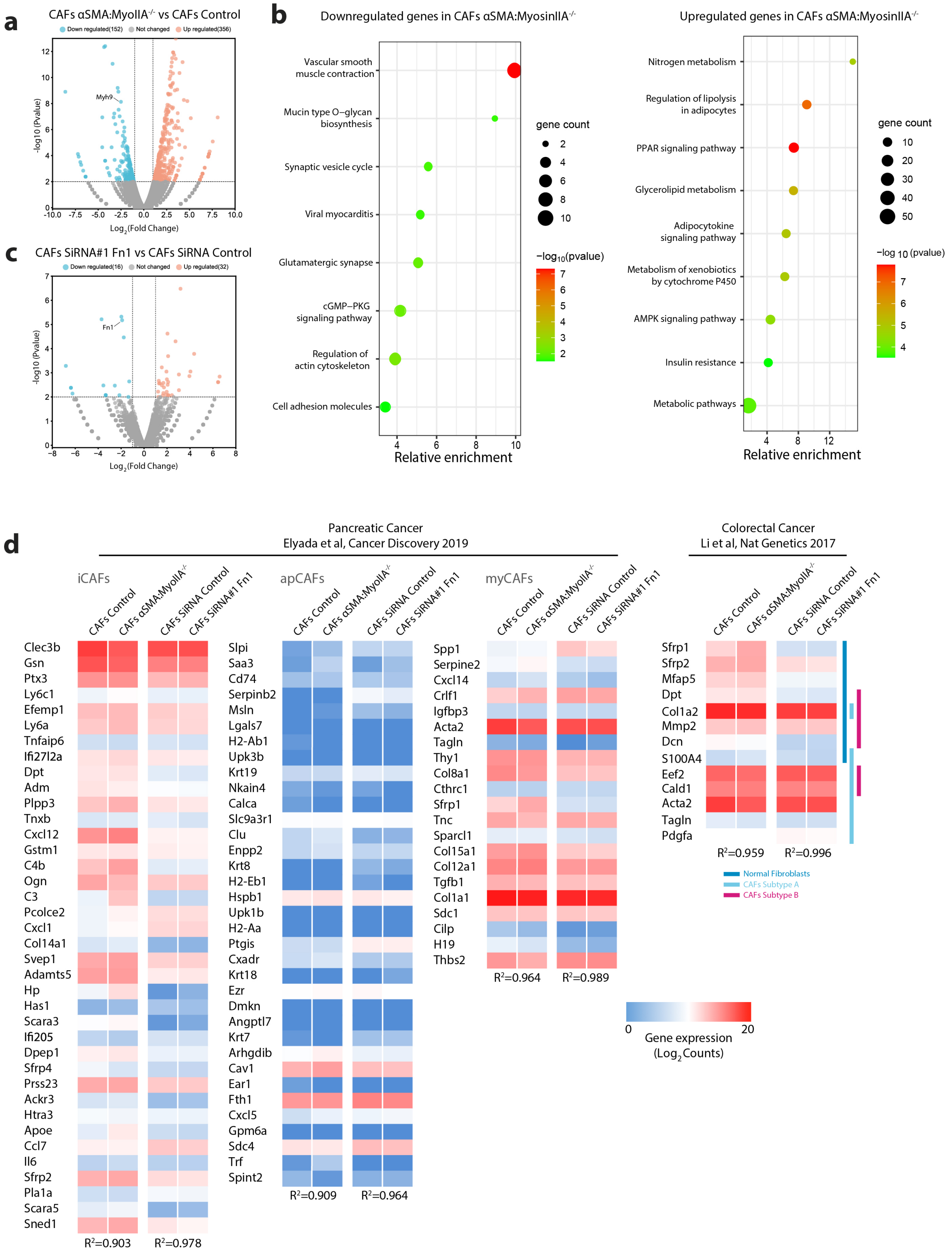
a
CAFs αSMA:MyoIIA-/- vs CAFs Control
Down regulated(152)
Not changed
Up regulated(356)
Myh9
-log10 (Pvalue)
Log2(Fold Change)
b
Downregulated genes in CAFs αSMA:MyosinIIA-/-
Vascular smooth muscle contraction
Mucin type O−glycan biosynthesis
Synaptic vesicle cycle
Viral myocarditis
Glutamatergic synapse
cGMP−PKG signaling pathway
Regulation of actin cytoskeleton
Cell adhesion molecules
Relative enrichment
gene count
−log10(pvalue)
Upregulated genes in CAFs αSMA:MyosinIIA-/-
Nitrogen metabolism
Regulation of lipolysis in adipocytes
PPAR signaling pathway
Glycerolipid metabolism
Adipocytokine signaling pathway
Metabolism of xenobiotics by cytochrome P450
AMPK signaling pathway
Insulin resistance
Metabolic pathways
Relative enrichment
gene count
−log 10 (pvalue)
c
CAFs SiRNA#1 Fn1 vs CAFs SiRNA Control
Down regulated(16)
Not changed
Up regulated(32)
Fn1
-log10 (Pvalue)
Log2(Fold Change)
d
Pancreatic Cancer
Elyada et al, Cancer Discovery 2019
Colorectal Cancer
Li et al, Nat Genetics 2017
iCAFs
apCAFs
myCAFs
CAFs Control
CAFs αSMA:MyoIIA-/-
CAFs SiRNA Control
CAFs SiRNA#1 Fn1
Clec3b Gsn Ptx3 Ly6c1 Efemp1 Ly6a Tnfaip6 Ifi27l2a Dpt Adm Plpp3 Tnxb Cxcl12 Gstm1 C4b Ogn C3 Pcolce2 Cxcl1 Col14a1 Svep1 Adamts5 Hp Has1 Scara3 Ifi205 Dpep1 Sfrp4 Prss23 Ackr3 Htra3 Apoe Ccl7 Il6 Sfrp2 Pla1a Scara5 Sned1
R²=0.903
R²=0.978
Slpi Saa3 Cd74 Serpinb2 Msln Lgals7 H2-Ab1 Upk3b Krt19 Nkain4 Calca Slc9a3r1 Clu Enpp2 Krt8 H2-Eb1 Hspb1 Upk1b H2-Aa Ptgis Cxadr Krt18 Ezr Dmkn Angptl7 Krt7 Arhgdib Cav1 Ear1 Fth1 Cxcl5 Gpm6a Sdc4 Trf Spint2
R²=0.909
R²=0.964
Spp1 Serpine2 Cxcl14 Crlf1 Igfbp3 Acta2 Tagln Thy1 Col8a1 Cthrc1 Sfrp1 Tnc Sparcl1 Col15a1 Col12a1 Tgfb1 Col1a1 Sdc1 Cilp H19 Thbs2
R²=0.964
R²=0.989
Sfrp1 Sfrp2 Mfap5 Dpt Col1a2 Mmp2 Dcn S100A4 Eef2 Cald1 Acta2 Tagln Pdgfa
R²=0.959
R²=0.996
Normal Fibroblasts
CAFs Subtype A
CAFs Subtype B
Gene expression (Log2 Counts)

Supplementary Data Figure. 12. Changes in gene expression and CAF subtypes upon Myosin IIA KO and Fibronectin KD.

a, Volcano plots showing the differentially expressed genes in Myosin IIA KO vs. control CAFs (thresholds, Fold Change = 2,-2; P value = 0.01). **b,** Bubble plot representing the main gene ontology terms (KEGG) enriched in downregulated (left) and upregulated (right) gene sets in CAFs Myosin IIA KO compared to control. **c,** Volcano plots of the differentially expressed genes in siRNA Fibronectin CAFs vs. SiRNA Control CAFs (thresholds, Fold Change = 2,-2; P value = 0.01). Fibronectin KD gene ontology is not shown because we considered the number of differentially expressed genes too low to perform gene ontology analyses. **d,** Expression of selected gene sets, previously defined as markers for different CAF subtypes in pancreatic (iCAFs, apCAFs and myCAFs, Eylada et al. 2019) (left), and colorectal cancer (normal fibroblasts, CAFs subtype A and CAFs subtype B, Li *et al.* 2017) (right), comparing control vs. Myosin KO CAFs, and SiRNA Control vs. Fibronectin-depleted CAFs. Correlations were tested by linear regression ($p<0.001$ for all comparisons).

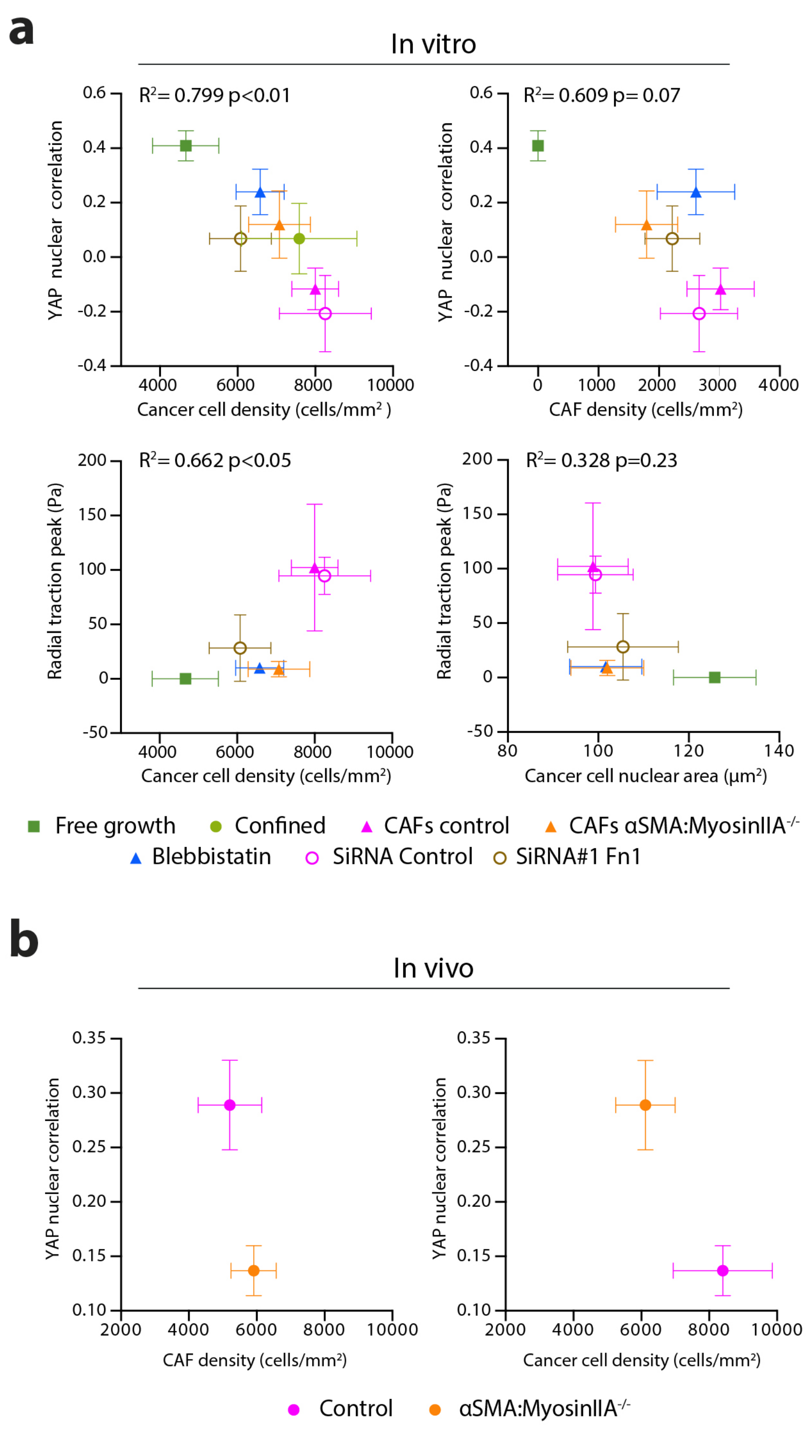

Supplementary Data Figure. 13. Correlation between mechanics, tissue organization and YAP localization.

a, Correlations between different parameters measured in compression assays in vitro: cancer cell density, CAF density, radial traction peak (readout of compression), nuclear area and YAP

nuclear correlation. **b,** Tumor CAF (left) and cancer cell (right) density vs. YAP nuclear correlation. Correlations were tested by linear regression.

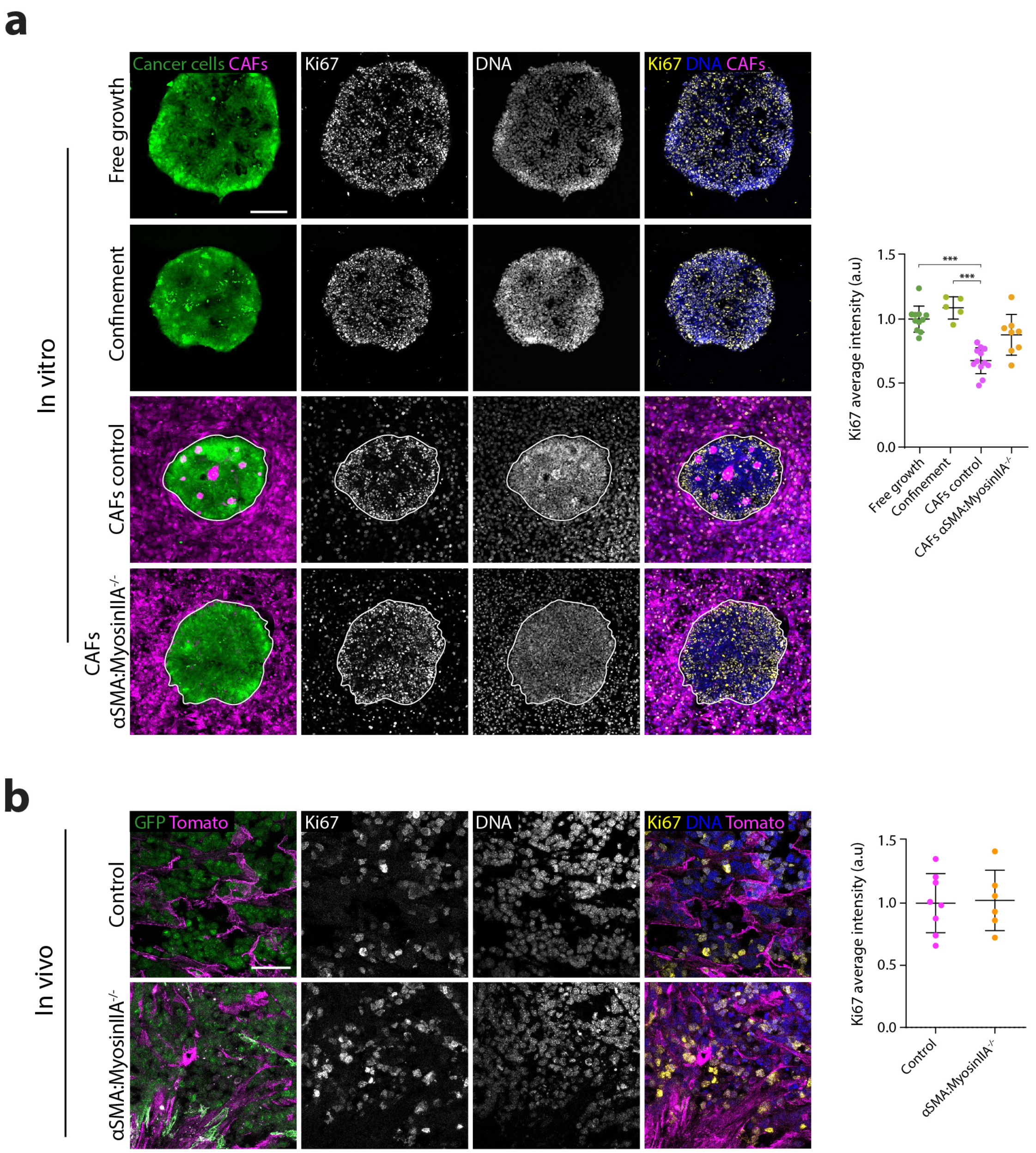

Supplementary Data Figure. 14. CAF compression decreases the number of cycling cancer cells.

a, Cancer cells (Cell tracker, Green) cultured on an adherent substrate where they grow freely, under confinement (PDMS stencils), or surrounded by control or Myosin IIA KO CAFs (Cell tracker, magenta). White lines represent the contour of the cluster of cancer cells. Cells are stained for Ki67 (yellow) and DNA (DAPI, blue). Graph: quantification of average Ki67 intensity in the nucleus of cancer cells, normalized to the free growth condition. Data represented as mean ± SD, n=5-12 clusters per condition, from N≥2 independent experiments. * $p<0.05$, ***

$p<0.001$, Kruskal-Wallis ANOVA test, Dunns multiple comparisons test. Scale bar: 100µm. **b,** Immunostainings of Ki67 (yellow) and DNA (DAPI, blue) on control and αSMA:MyosinIIA$^{-/-}$ tumors. Cancer cells express nuclear GFP (green), the stroma is labeled in magenta and αSMA:MyosinIIA$^{-/-}$ cells express membrane GFP (green). Graph: quantification of average Ki67 intensity in the nucleus of cancer cells. Data represented as mean ± SD. Each dot represents the average of 10 image quantifications per tumor. n=8 (control) and n=6 (αSMA:MyosinIIA$^{-/-}$). Scale bar: 50µm.

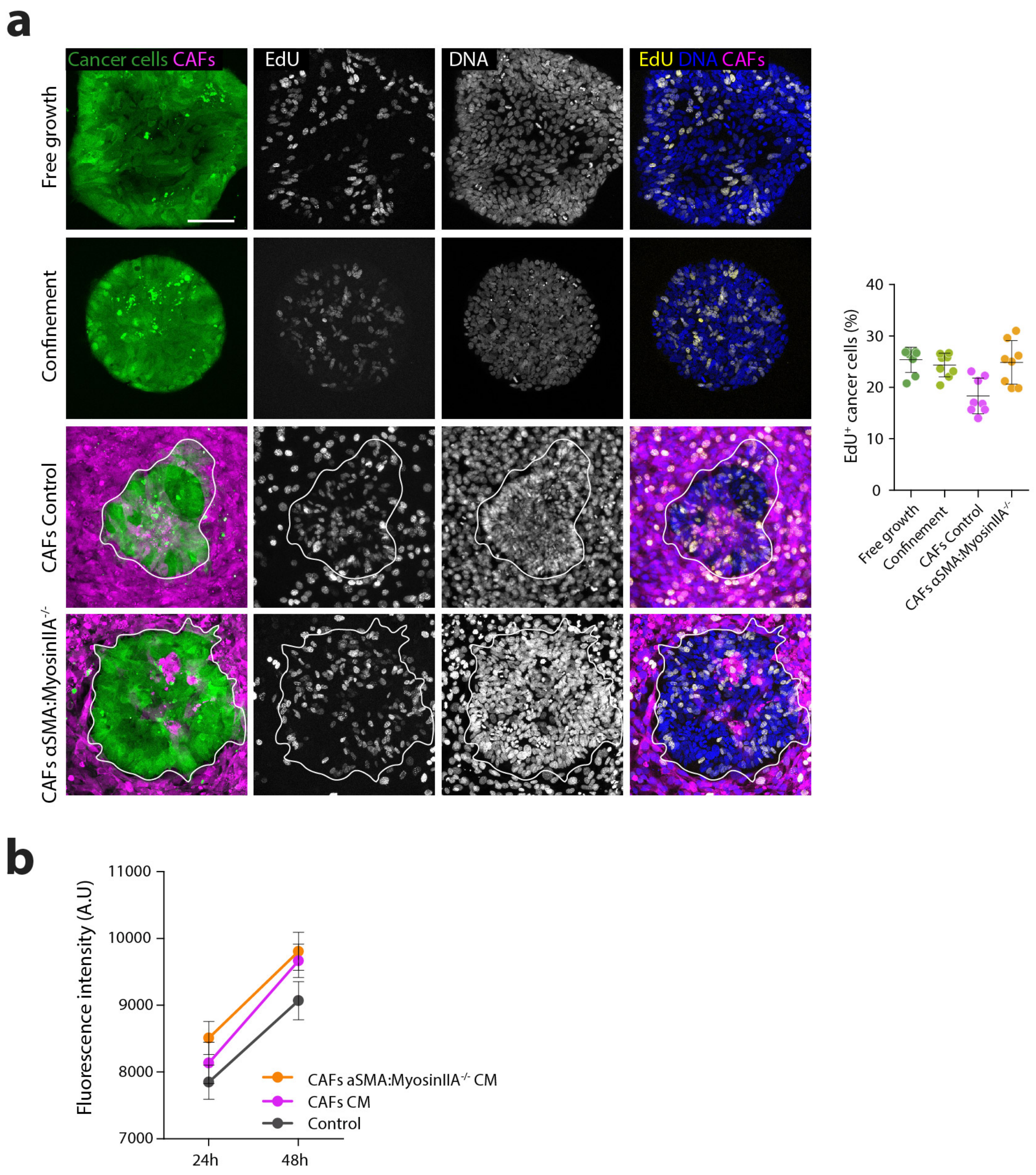

Supplementary Data Figure. 15. CAF compression decreases the number of cancer cells in S-phase.

a, Cancer cells (Cell tracker, Green) cultured on an adherent substrate where they grow freely, under confinement (PDMS stencils), or surrounded by control or Myosin IIA KO CAFs (Cell tracker, magenta). White lines represent the contour of the cluster of cancer cells. A 60 minutes pulse of EdU was performed before fixation to label cells in S-phase. Cells were then stained for incorporated EdU (yellow) and DNA (DAPI, blue). Graph: percentage of EdU^+ cells in the cancer cell cluster. Data represented as mean ± SD, n=8 clusters per condition, from N=2 independent experiments. Scale bar: 100 µm. **b,** Fluorescence intensity as a readout of

proliferation in cancer cells treated with control or conditioned medium from control and MyosinIIa KO CAFs from N=2 independent experiments.

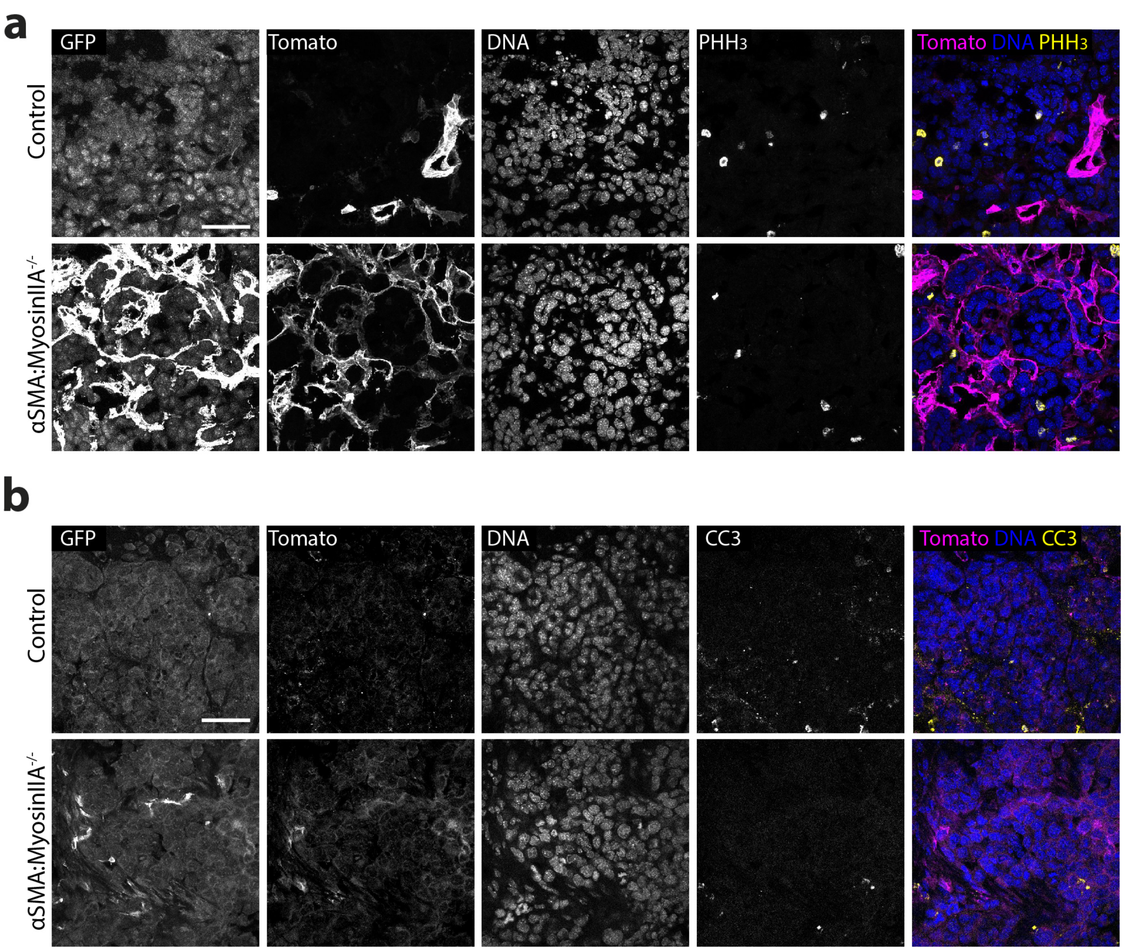

Supplementary Data Figure. 16. Phospho-histone H3 and Cleaved-caspase 3 immunostainings in tumors.

a-c. Representative immunostainings of Phospho-histone H3 (yellow) (**a**) and Cleaved-caspase 3 (yellow) (**b**) in control and αSMA:MyosinIIA$^{-/-}$ subcutaneous tumors. Cancer cells express nuclear GFP (green), the stroma expresses mTomato (magenta) and αSMA:MyosinIIA$^{-/-}$ cells express membrane-tagged GFP (green). DNA is labeled with DAPI (blue). Images and mice numbers correspond to quantifications of panels c and e of Figure 6. Scale bar: 50 µm.

Supplementary Note 1

In this note, we develop a physical model of the remodeling of the cancer cell cluster (CC cluster) by Cancer Associated Fibroblasts (CAFs) in the *in vitro* co-culture system. We aimed at providing a unified framework to quantitatively described the closure kinetics and the associated traction forces measured on the substrate, and to relate these to the pillar compression experiments.

S1 Model

A sketch of the model system is presented in **Supp. Fig. 1a**. The CC cluster constitutes a cell monolayer prior to CAF remodeling. The closure of the CAF monolayer on top of the cluster is assumed to be solely driven by the contraction of the acto-myosin ring present at the inner edge of the CAFs, modeled as a constant line tension Γ [1]. We concentrate on the dynamics characterized by inward traction forces, for which CAF migration may be disregarded. We further assume radial symmetry, which, although not particularly accurate in experimental situations such as the one shown in **Fig. 1d** of the main text, is a prerequisite for an analytically tractable model.

We adopt a thin sheet approximation for the mechanical description of the plane stress tensor γ_{ij} in the CAF monolayer, and the stress tensor in the CC cluster, for which we only consider the integrated version over cluster thickness $h\sigma_{ij} \equiv \int_0^h \mathrm{d}z\, \sigma_{ij}$.

Several spatial regions must be distinguished, characterised by the radius of the CAF inner edge (the acto-myosin ring) R_c and the outer edge of the CC cluster R_T (see sketch on **Supp. Fig. 1a**).

- For $r > R_T$, the CAFs are in direct contact with the substrate and experience a substrate force density T_r. The local force balance in this region reads [2]:

$$\partial_r \gamma_{rr}(r) + \frac{\gamma_{rr}(r) - \gamma_{\theta\theta}(r)}{r} = T_r(r). \tag{S1}$$

  where γ_{rr} and $\gamma_{\theta\theta}$ are the radial and orthoradial components of the CAF stress tensor and r is the radial coordinate.

- For $R_c + \delta < r < R_T$, where δ is the width of the actomyosin ring, the CAFs are in contact with the CC cluster. The sliding of the CAFs on top of the CC cluster creates a shear stress $f(r)$ which acts as a source of tension gradient in both CAFs and CC cluster:

$$\partial_r \gamma_{rr}(r) + \frac{\gamma_{rr}(r) - \gamma_{\theta\theta}(r)}{r} = f(r)\ ,\ \partial_r h\sigma_{rr}(r) + \frac{h\sigma_{rr}(r) - h\sigma_{\theta\theta}(r)}{r} = -f(r) + T_r(r) \tag{S2}$$

- For $R_c < r < R_c + \delta$, the balance between the inward force from the ring line tension Γ/R_c and the CAF radial tension may involve a particular lineic friction force f_Γ, which in turn causes a discontinuity $\delta h\sigma_{rr}$ in the CC cluster stress :

$$\gamma_{rr}(R_c) - \frac{\Gamma}{R_c} = f_\Gamma\ ,\ \delta h\sigma_{rr} = -f_\Gamma + T_r(R_c)\delta \tag{S3}$$

- For $r < R_c$, the central part of the CC cluster is not covered by the CAFs. Prior to bud formation, the central cluster is weakly deformed and does not directly exert stress on the actomyosin ring. The force balance in the CC cluster simply reads

$$\partial_r h\sigma_{rr}(r) + \frac{h\sigma_{rr}(r) - h\sigma_{\theta\theta}(r)}{r} = T_r(r) \tag{S4}$$

The persistence of inward pointing traction forces after the ring has reached a stationary position (**Fig. 1e**, main text, reproduced in **Supp. Fig. 1b**) suggests an elastic description for the mechanics of CAFs and CC cluster, and an elastic-like interaction with the substrate[1, 2]. For a 3D-incompressible elastic description of the CAF monolayer characterised by a 2D elastic modulus E_c, the components of the stress tensor are related to the radial displacement $u(r)$ in the CAF layer (where r is the radial coordinate) according to:

$$\gamma_{rr}(r) = \gamma_c + \frac{2}{3}E_c\left[2\partial_r u(r) + \frac{u(r)}{r}\right] \;,\; \gamma_{\theta\theta}(r) = \gamma_c + \frac{2}{3}E_c\left[2\frac{u(r)}{r} + \partial_r u(r)\right] \tag{S5}$$

where γ_c is the homeostatic contractile tension in the CAF monolayer far from the ring. Further assuming an elastic interaction with the substrate: $T_r(r) = Y_s u(r)$ where Y_s is a static friction coefficient, the solution of Eq. 1 for the CAF displacement in the region $r > R_T$ is $u(r) \propto K_1(r/\lambda_s)$ where K_1 is a modified Bessel function, which leads to a spatial localisation of the substrate stress over a characteristic length scale $\lambda_s \equiv \sqrt{4E_c/(3Y_s)}$ [2]. This analytic solution is likely to be of limited validity in the present context, as the tissue mechanics is more complex than a simple elastic sheet, and include cellular rearrangements, especially during bud formation. However, the localisation of the traction forces outside the CC cluster is indeed observed (with $\lambda_s \simeq 50$ μm), which suggests that the spatial derivative of the stress dominate the RHS of Eqs. 1,2,4 (quasi 1D approximation). These three local force balances can be integrated and added to Eq. 3 to obtain the global force balance

$$\frac{\Gamma}{R_c} = \gamma_c - \int_0^\infty T_r(r)dr - \gamma_{\text{bud}} \qquad \gamma_{\text{bud}} = h\sigma_{rr}|_{r=0} \tag{S6}$$

The LHS of this equation represents the force (per unit length) driving CAF closure and the RHS the forces resisting closure, which includes the CAF homeostatic tension, substrate friction, and the compression of the central region of the CC cluster. The expression given for γ_{bud} corresponds to the low deformation limit of Eqs.1-4. However, Eq. 6 remains valid when a tumor bud forms in the central region tof the CC cluster, provided the bud compressive stress $-\gamma_{\text{bud}}$ is calculated by taking into account cellular rearrangement in the bud (see below).

S2 Traction force and closure dynamics

Eq. 6 may be seen as a dynamic equation for the evolution of the ring radius $R_c(t)$ as all the terms present in it (apart from γ_c) depend on time. The LHS of Eq. 6 clearly increases with time as the radius shrinks, so must the RHS. We may assume that the CAF tension far from the cluster γ_c remains constant over time, so the increase of the closure force must be compensated by either an increase of traction force or by an increase of the bud resistance to compression or by both. **Supp. Fig. 1c,d** shows the temporal evolution of the total traction force [1] and the average ring radius (obtained from the ring area shown **Fig. 1f** in the main text). Two dynamical regimes can be distinguished from the moment where the total traction force points inward (**Supp. Fig. 1d**). At earlier time (from 7 to 16 hrs), the closure dynamics is fast ($dR_c/dt = -3.3$ μm/h) and corresponds to a linear increase of the inward traction force with time. At later time (16 to 27 hrs), the closure dynamics is slower ($dR_c/dt = -1$ μm/h) and the traction force saturates to a value $R_c \int_0^\infty T_r dr = 1.6\mu$N.

The linear increase of inward traction force at short time is consistent with the increase of the driving force from the line tension Γ/R_c as the ring closes, although part of the increase of inward traction force must probably also be attributed to the decrease of active outward

[1] Two alternative definitions: $\int \mathrm{d}r\, rT_r$ and $R_c \int \mathrm{d}r\, T_r$, give similar results (**Supp. Fig. 1c**) which supports the approximation of localised tractions made to obtain Eq. 6.

traction force associated to CAF migration. The fact that the traction force saturates while the ring is still closing is remarkable, and indicates a build-up of stress in the central cluster region that is eventually driving bud formation. In this regime, only a fraction of the driving force is compensated by the traction force, which, at their saturation value, amounts to an effective line tension $\Gamma_s \simeq 1.2 - 1.6\mu$N. The remaining driving force must be compensated by the compression of the CC cluster. Thus, the CC cluster budding has a signature both in the closure dynamics and in the evolution of the traction force.

We can attempt to relate this value of the line tension to the stress measured in the pillar compression experiment reported in **Fig. 4** - main text. We see from **Fig. 4f** that the normal stress on pillars of radius $R_p = 50$ μm reaches $\sigma_p = 10^3$ Pa. This corresponds to a typical lateral tension in the CAF layer surrounding the pillar $\gamma_\mathrm{p} = \sigma_\mathrm{p} \mathrm{R_p} = 5 \times 10^{-2}$ N/m. Multiplying γ_p by the typical thickness $h_c \simeq 10$ μm of the CAF monolayer yields an estimate for the line tension $\Gamma \simeq 500$ nN. This is slightly smaller but of the same order of magnitude as the estimate obtained from the traction force in the co-culture experiments. This shows that the CAFs are indeed able to exert a large compressive stress, when compared, for instance, to the typical tension of one stress fiber, which is of order 10 nN [3].

S3 Bud stability

The cancer cell buds remain stable over time (**Fig. 1e,f** - main text and **Supp. Fig. 1b**) when directly compressed by the CAF ring. This experimental observation is not straightforward from a theoretical perspective. Indeed, the cellular rearrangements and multilayering involved in bud formation suggests a stress-dependent fluidification of the CC cluster. If the cluster behaved as a fluid, the bud would adopt a spherical shape due to its apical tension γ_{cc}. Experimental observations show that buds are rather cylindrical with small height-to-diameter ratio (**Extended Data Fig. 4b,c**). Furthermore, the stability of such fluid bud pinched by the actomyosin ring would rely on the ratio of driving to resisting force $\Gamma/(\gamma_{cc} R_c)$ to be of order unity. With a typical CC apical tension $\gamma_{cc} \sim 10^{-3}$ N/m and with $R_c \simeq 100$ μm, this ratio is about 10 times smaller.

To simplify the discussion of bud stability, we neglect the traction forces which saturate to a constant value after bud formation. We describe the bud as a stratified structure composed of n layers (**Supp. Fig. 1e**), and we only discuss the diagonal component of the stress tensor in each layer, defined as a pressure P. To account for the plastic cellular rearrangements within the bud, we introduce a yield pressure P^* that describes phenomenologically the amount of compressive stress necessary to trigger cell exchange between layers. CAF closure initially occurs on a monolayer of CCs, and force transmission through friction ($f(r)$ and f_Γ in Eqs.2,3) leads to the generation of a compressive stress P_1 in the central part of the CC monolayer. If the pressure remains below the threshold ($P_1 < P^*$), no multilayering occurs and the CAFs are able to close on top of the CC monolayer, as it is sometimes observed in our experiments (data not shown). If the threshold is exceeded, cells delaminate from the central region of the CC cluster and form a second layer. The particular ring radius at which this happens is defined as R_c^*. The pressure $P_2(t)$ in the second layer increases as the ring closes and cells are transferred from the first layer. If $P_2 > P^*$, a third layer forms, with pressure $P_3(t)$, and cells are expelled from the second payer until $P_2 - P_3 < P^*$. Only the second layer is directly compressed by the ring, so the bud resistive force in Eq. 6 is $\gamma_\mathrm{bud} = -hP_2$. The pressure P_2 is estimated as follows. For $P_2 < P^*$, $P_2 = E_{cc}(R_2^* - R_c)/R_2^*$, where E_{cc} is the bulk modulus of the layer and R_2^* is the ring radius at which layer 2 starts being compressed (**Supp. Fig. 1f**). For $P_2 > P^*$, $P_2 = P^* \phi(h_b/h)$, where h_b is the height of the bud, determined by volume conservation ($h_b = h(R_2^*/R_c)^2$) and $\phi(x)$ is a phenomenological shape function capturing the shear stress between adjacent layers. In analogy with the standard problem of elastic pillar

deformation induced by a collar pressure [4], we adopt the function $\phi(x) = 1 + b \tanh[(x-1)/b]$ which appropriately describes the localisation of the stress near the ring for tall bud ($h_b \gg h$), as seen in the pillar compression experiment (**Fig. 4g** - main text). Here, b is a phenomenological parameter representing shear resistance between adjacent layers. For an homogeneous elastic material, finite element simulations yield $b \simeq 0.3$. In the present context, b is determined by cell-cell adhesion and can be considered as a free parameter.

Supp. Fig. 1f shows a graphical representation of the static force balance between the ring tension and the bud compression $\Gamma/R_c = \gamma_c + hP_2$. The existence of a static equilibrium with a finite ring radius state crucially depends on the parameter b. If the ring tension is shared by all layers ($b \to \infty$), there is always a single stable equilibrium as the height of the bud diverges when R_c vanishes (solid gray line in **Supp. Fig. 1f**). In the opposite limit ($b \to 0$) different layers are uncoupled, and $P_2 \leq P^*$. Since the formation of a second layer requires $\Gamma/R_c > \gamma_c + hP^*$, no static equilibrium exists at finite R_c and multilayering necessarily leads to ring closure below the bud (dashed gray line in **Supp. Fig. 1f**). Under more realistic situations, bud compression leads to a maximum resistive force $hP^*(1+b)$ that defines three phenotypes according to the parameter values (**Supp. Fig. 1e,f**): full budding and CAF closure, stable bud with a finite neck size and CAF closure with no bud. This is summarized on **Supp. Fig. 1g** with a phase diagram as a function of reduced yield pressure P^*/E_{cc} and reduced line tension $\Gamma/(E_{cc}h^2)$. If P^* is small, the CAF shear stress triggers the multilayering transition for large ring radius but the maximum bud resistive force is too small to equilibrate the ring compression. If P^* is large, the multilayering transition occurs for a small ring radius, for which ring compression increases faster than the bud resistive force with decreasing ring radius. In both cases, cells are expelled from the second layer until the CAF ring closes. For even larger P^*, multilayering would only occur for a ring radius of order the cell size, in which case we consider that the ring closes without bud formation (gray region on **Supp. Fig. 1g**). Thus, bud stability occurs only on a parametric region were both ring line tension and yield pressure are sufficiently small, and can be enhanced with an increase of CC elastic modulus E_{cc} or effective parameter b. From the estimation $\Gamma \sim 1$ μN obtained in the previous part and with $h \sim 10$ μm, stable buds require $E_{cc} \gtrsim 1$ kPa and $P^* \simeq 0.1 - 0.4E_{cc}$. Thus, CAF compression around an incomplete CC bud provides an indirect way to measure the tissue elastic modulus, found here to be consistent with typical values measured for cancer cells [5].

References

[1] S. R. K. Vedula, G. Peyret, I. Cheddadi, T. Chen, A. Brugués, H. Hirata, H. Lopez-Menendez, Y. Toyama, L. Neves de Almeida, X. Trepat, C. T. Lim, and B. Ladoux, "Mechanics of epithelial closure over non-adherent environments," *Nature Communications*, vol. 6, no. 1, p. 6111, 2015.

[2] C. M. Edwards and U. S. Schwarz, "Force Localization in Contracting Cell Layers," *Phys. Rev. Lett.*, vol. 107, no. 12, p. 128101, 2011.

[3] S. Deguchi, T. Ohashi, and M. Sato, "Tensile properties of single stress fibers isolated from cultured vascular smooth muscle cells," *Journal of Biomechanics*, vol. 39, no. 14, pp. 2603–2610, 2006.

[4] V. V. Meleshko and Y. V. Tokovyy, "Equilibrium of an elastic finite cylinder under axisymmetric discontinuous normal loadings," *J Eng Math*, vol. 78, no. 1, pp. 143–166, 2013.

[5] C. Alibert, B. Goud, and J.-B. Manneville, "Are cancer cells really softer than normal cells?," *Biology of the Cell*, vol. 109, no. 5, pp. 167–189, 2017.

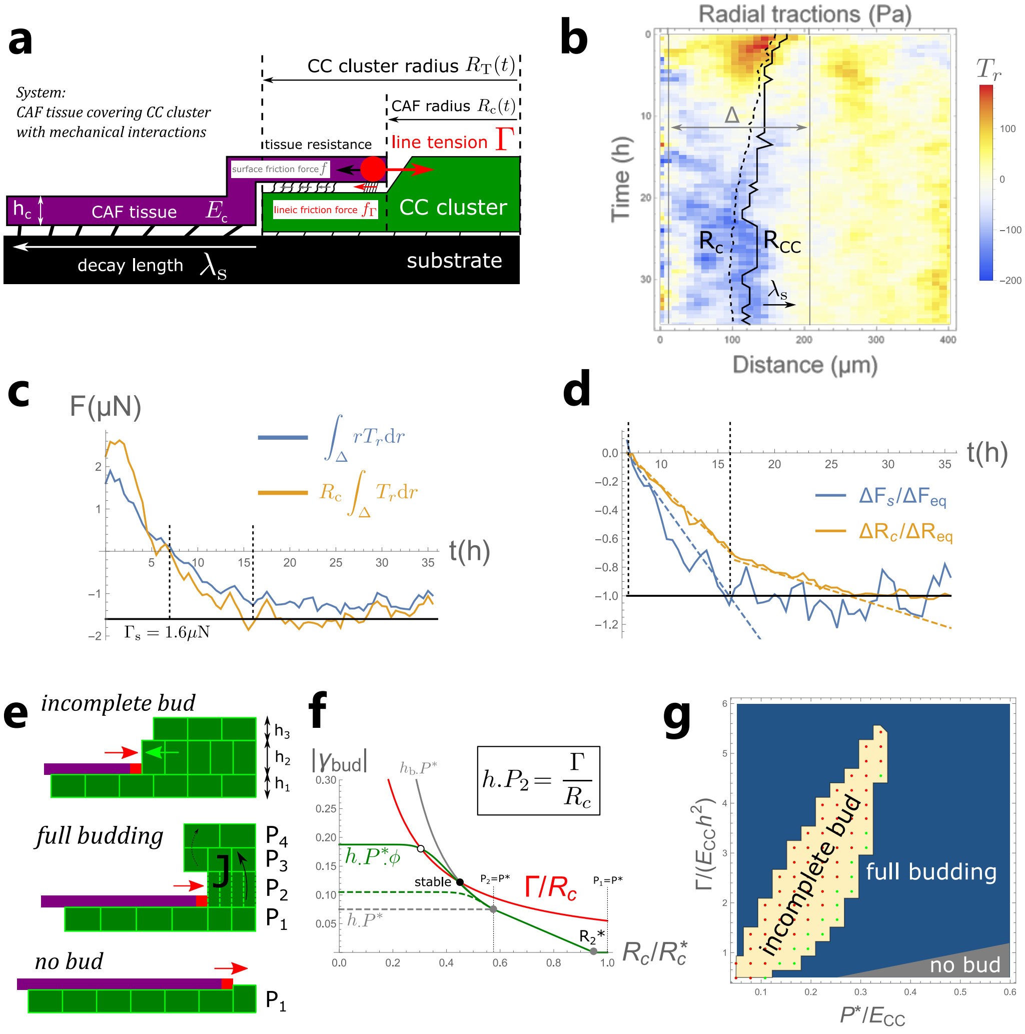

Supplementary note figure 1 **(a)**: Sketch of the model: a CAF monolayer (purple) closes on top of a cluster of cancer cells (green), driven by the line tension provided by a dense actomyosin ring (red). The CAFs exert a surface friction force on the CC cluster – which may include a lineic friction f_Γ at the location of the ring – that creates cluster compression. The movement of both the CAFs and the CC cluster generate traction forces on the substrate. Within an elastic model, the traction force below the CAF layer are localised within a decay length λ_s. **(b)**: Experimental kymograph of substrate tractions T_r (reproduced from **Fig. 1e** - main text) showing the localization on length λ_s. Dashed and solid black lines respectively represent the location of the actomyosin ring and CC cluster periphery. We only focus on times for which tractions are inward (blue). The ROI is defined as the spatial range Δ (gray lines). Outside this range, numerical ($r = 0$) or biological ($r > 200$ µm) fluctuations are dominant. **(c)**: The computed total traction force over the ROI, through a surface integral $\int_\Delta rT_r(r)\mathrm{d}r \equiv F_s/(2\pi)$ (blue) or a line integral $R_c\int_\Delta T_r(r)\mathrm{d}r$ (orange). Inward tractions (negative) start after $t = 7$ h and reach a saturation value between 1.2 and 1.6 µN for $t > 16$ h. **(d)**: Relative variation of the total traction force and the ring area from their value at $t = 7$ h, normalised by their saturation value at long time. A striking feature is the apparent saturation of traction force before CAF edge radius saturation, that we interpret as an indication of bud compression. For all studied clusters with stabilized buds, abrupt changes in ring and traction dynamics coincide. **(e)**: Sketch of the plastic model of bud formation: CC cluster multilayering occurs if the stress within the cluster generated by CAF closure exceeds a threshold P^*, and cell transfer between adjacent layers occurs if the stress difference between the two layers exceed P^*. The second CC layer directly resists the CAF ring tension. This may result in a stable incomplete bud with a finite neck size, or ring closure and full budding. **(f)**: Force balance between the CAF ring compression (red) and the bud elastic resistance (green), as a function of ring radius R_c. The shear-transmitted resistance from adjacent layers to second layer is captured by a shape factor $\phi = 1 + b\tanh[(h_b/h - 1)/b]$. A second layer is formed for a particular ring radius $R_c = R_c^*$ (when $P_1 = P^*$), and is compressed up to $P_2 = P^*$ and eventually builds more layers. This may lead to a stable incomplete bud (green) or to ring closure and full budding (dashed green) if bud resistance is too weak. The parameter b characterises the shear stiffness between layers. The dashed and solid gray lines show the asymptotic cases $b \to 0$ and $b \to \infty$, respectively. **(g)**: Budding phase diagram as a function of the dimensionless yield pressure P^* and ring line tension Γ (E_{cc} is the CC bulk modulus). A stable bud occurs only for a limited range of both parameters (yellow) and can contain two layers (green dots) or more than two (red dots). The "no bud" region (gray) corresponds to a bud that would contain less than two cells. Parameters used are $E_c = 0$, $b = 1.5$.
