# Supplementary material for: Cancer-associated fibroblasts actively compress cancer cells and modulate mechanotransduction
Source: Nat Commun. 2023 Nov 1;14:6966. doi: 10.1038/s41467-023-42382-4 (PMC10618488; doi:10.1038/s41467-023-42382-4)
Supplement: Supplementary file 2 — Description of Additional Supplementary Files [file 41467_2023_42382_MOESM2_ESM.pdf]

Description of Additional Supplementary Files

Supplementary Video 1. CAFs form a 3D capsule around cancer cells in tumors.

3D image of N/p53 tumor slice showing the organization of cancer cells (mGFP, green) and stroma (mTomato, magenta). The stroma forms intratumoral capsules that surround cancer cells and compartmentalize the tumor in clusters, as shown in the orthogonal views. Cell nuclei are labeled by DAPI (blue).

Supplementary Video 2. CAFs compress cancer cells in vitro.

<u>CAFs compression in cocultures</u>: Top (top) and lateral (bottom) views of the in vitro co-culture system showing a cluster of cancer cells (isolated from PDX, green) surrounded by CAFs (magenta). CAFs form a ring that closes on top of the cancer cells, reshaping the cluster and inducing the formation of a three-dimensional bud. White line indicates contour of the CAFs ring. Yellow line labels the location of the orthogonal view. Scale bar, 100 µm. Time, hh:mm.

<u>Imaris rendering of a 48h coculture</u>: 3D rendering of the CAFs-cancer cell coculture when the bud is stabilized (t=48h). Note that the CAFs ring is stalled around the bud, compressing cancer cells.

Supplementary Video 3. Coculture of mouse cancer cells and CAFs.

Phase contrast (left) and fluorescence of N/p53 cancer cells (right, mGFP) showing the evolution of the coculture. CAFs surround, reshape and compress cancer cells. Scale bar, 100 µm. Time, hh:mm.

Supplementary Video 4. Spontaneous organization of CAFs and cancer cells from PDX tissue fragments

Timelapse (phase contrast) of a coculture of cancer cells and CAFs that spontaneously exited a PDX tissue fragment. CAFs surrounded and confined cancer cells (right cluster). Some CAFs assemble a supracellular ring that reshapes and compresses cancer cells (bottom left cluster, yellow line). Scale bar, 100 µm. Time, hh:mm.

Supplementary Video 5. Traction forces in cocultures

Evolution of traction forces during the reshaping of the cancer cell cluster by CAFs. Left, DIC movie of the coculture outlining the contour of the cancer cell cluster (solid red line) and the CAFs ring (dashed red line). Right, evolution of traction forces exerted by CAFs and cancer cells (black line labels the cancer cell cluster boundary). Tractions accumulate at the interface between cancer cells cluster and CAFs. At first timepoints, tractions point away from the cluster. When the CAFs ring is formed (t=9:00h) and during cluster reshaping, tractions point towards the cluster. Scale bar, 100 µm. Time, hh:mm.

Supplementary Video 6. Inhibition of contractility suppresses compression of cancer cells in vitro

Left, evolution of traction forces after treatment with blebbistatin. Right, evolution of traction forces after treatment with Y27632. Treatment starts at t=0h. Solid red line indicates the contour of the ring, dashed red line indicates the position of the ring immediately before treatment. Both treatments induce a massive decrease in traction forces and a fast relaxation of the CAFs ring. Scale bar, 100 µm. Time, hh:mm.

Supplementary Video 7. Tissue displacements after laser ablation in tumors and in vitro cocultures

Left: laser ablation (t=0s) of the boundary between cancer cells (green) and the stroma (magenta) in a tumor slice. Ablated area is indicated in white. Tissue displacements are represented by white vectors. After ablation, cancer cells move towards the cut, showing that they are compressed. Solid cyan lines indicate the ROIs far from cut and near cut quantified in figure 2B. The cancer cell cluster is outlined by a dashed cyan line. Scale vector: 1 µm.

Center: laser ablation (t=0s) of the stroma (magenta) perpendicular to the boundary with cancer cells (green) in a tumor slice. Ablated area is indicated in white. Tissue displacements are represented by white vectors. After ablation, cancer cells move towards the cut, showing they are compressed, and the stroma recoils away from the cut, showing it is under tension. Solid cyan lines outline the ROIs of the stroma and cancer cells quantified in figure 2D. Yellow vectors show the average displacement in each ROI (for visualization purposes they are not scaled). The cancer cell cluster is outlined by a dashed cyan line. Scale vector: 1 µm.

Right: laser ablation (t=0s) of CAFs (magenta) perpendicular to the boundary with cancer cells (green) in vitro. Ablated area is indicated in white. Tissue displacements are represented by white vectors. After ablation, cancer cells move towards the cut, showing they compressed, and CAFs recoil away from the cut, showing they are under tension. Solid cyan lines outline the ROIs of the CAFs and cancer cells quantified in Fig. 2F. Yellow vectors show the average displacement in each ROI (for visualization purposes they are not scaled). The cancer cell cluster is outlined by a dashed cyan line. Scale vector: 5 µm. Scale bar, 100 µm. Total time, 50 seconds.

Supplementary Video 8. Tissue displacements after laser ablation in tumors with impaired CAFs contractility

Laser ablations (t=0s) of the stroma perpendicular to the boundary with cancer cells (nGFP, green) in a control tumor (left), a blebbistatin treated tumor (center) and a tumor containing Myosin IIA KO CAFs (right). The stroma is labeled in magenta (mTomato) with the exception of Myosin IIA KO CAFs, which became green (mGFP) after myosin depletion. Ablated area is indicated in white. Tissue displacements are represented by white vectors. In the control tumor, cancer cells move towards the cut, showing they are compressed, and the stroma recoils away from the cut, showing it is under tension. This recoil pattern is lost when contractility is inhibited either by blebbistatin or by knocking-out myosin IIA in CAFs. Solid cyan lines outline the ROIs of the stroma and cancer cells quantified in figure 2G. Yellow vectors show the average displacement in each ROI (for visualization purposes they are not scaled). Cancer cell clusters are outlined by a dashed cyan line. Scale vector: 1 µm. Total time, 50 seconds.

Supplementary Video 9. 3D visualization of a polyacrylamide pillar and CAFs organization

<u>3D pillar visualization:</u> 3D rendering of fluorescent beads embedded in the polyacrylamide gel to visualize pillar 3D shape.

<u>3D visualization of CAFs in pillars:</u> CAFs organization around the pillar, visualized by immunostaining of F-actin (magenta), phospho-Myosin Light Chain (green) and nuclei (DAPI). CAFs form supracellular stress fibers rich in active myosin and align parallel to the pillar surface to compress it.

Supplementary Video 10. CAFs traction forces exerted on the pillar

Top (left) and side (right) view of a 3D pillar model in a relaxed state (frame 1), and progressively showing the deformations (orange) induced by CAFs traction forces (black vectors). Note that deformation is magnified 5 times for visualization purposes.

Supplementary Video 11. Traction forces in cocultures of control and fibronectin-depleted CAFs

Time-lapse of cocultures with control (top) and fibronectin-depleted (bottom) CAFs. Left, DIC image of the coculture outlining the contour of the cancer cell cluster (solid red line) and the CAFs ring (dashed red line). Right, evolution of traction forces exerted by CAFs and cancer cells (black line labels the cancer cell cluster boundary). Depletion of fibronectin impairs CAFs traction forces, ring formation and compression of the cancer cell cluster. Scale bar, 100 µm. Time, hh:mm.
